# Chemical isotope labeling for quantitative proteomics

**DOI:** 10.1002/mas.21709

**Published:** 2021-06-06

**Authors:** Xiaobo Tian, Hjalmar P. Permentier, Rainer Bischoff

**Affiliations:** ^1^ Department of Analytical Biochemistry and Interfaculty Mass Spectrometry Center, Groningen Research Institute of Pharmacy University of Groningen Groningen The Netherlands

**Keywords:** fragment ion, isobaric labeling, quantitative proteomics, stable isotope labeling, tandem mass spectrometry

## Abstract

Advancements in liquid chromatography and mass spectrometry over the last decades have led to a significant development in mass spectrometry‐based proteome quantification approaches. An increasingly attractive strategy is multiplex isotope labeling, which significantly improves the accuracy, precision and throughput of quantitative proteomics in the data‐dependent acquisition mode. Isotope labeling‐based approaches can be classified into MS1‐based and MS2‐based quantification. In this review, we give an overview of approaches based on chemical isotope labeling and discuss their principles, benefits, and limitations with the goal to give insights into fundamental questions and provide a useful reference for choosing a method for quantitative proteomics. As a perspective, we discuss the current possibilities and limitations of multiplex, isotope labeling approaches for the data‐independent acquisition mode, which is increasing in popularity.

**Abbreviations**
Ac‐AG: acetyl‐alanine‐glycine
Ac‐IPG: acetyl‐isoleucine‐proline‐glycine
cICAT: cleavable isotope‐coded affinity tag
CILAT: cleavable isobaric labeled affinity tag
CMT: combinatorial isobaric mass tag
DDA: data‐dependent acquisition
DIA: data‐independent acquisition
DiART: deuterium isobaric amine reactive tag
DiLeu: N,N‐dimethyl leucine
FAIMS: high‐field asymmetric waveform ion mobility spectrometry
FT‐ICR: Fourier transform ion cyclotron resonance
IBT: isobaric tags
ICAT: isotope‐coded affinity tag
iTRAQ: isobaric tags for relative and absolute quantitation
IPTL: isobaric peptide termini labeling
LC‐MS: liquid chromatography mass spectrometry
MDHI: 2‐methoxy‐4,5‐dihydro‐1*H*‐imidazole
NCE: normalized collision energy
NHS: N‐hydroxysuccinimide
RP‐HPLC: reversed‐phase high‐performance liquid chromatography
SA: succinic anhydride
SILAC: stable isotope labeling by amino acids in cell culture
SMD‐IPTL: selective maleylation‐directed isobaric peptide termini labeling
SWATH‐MS: sequential windowed acquisition of all theoretical fragment ion mass spectra
S/N: signal‐to‐noise
TMT: tandem mass tags

## 1 INTRODUCTION

Proteomics aims at the comprehensive identification and quantification of all proteins in a biological system, to reveal the roles of proteins in biological, physiological, and pathological processes. Proteomics data quality has been continuously improving in terms of accuracy and precision while throughput has increased leading to an ever‐increasing flow of data. This is due to improvements of speed, sensitivity and resolution in liquid chromatography mass spectrometry (LC‐MS) as well as advances in data processing that benefitted from the increase in computational power and advanced algorithms. Proteomics is widely applied in many research areas, such as the exploration of biological mechanisms (Aebersold & Mann, 2016; Larance & Lamond, 2015), the discovery of biomarkers (Anjo et al., 2017; Borrebaeck, 2017; Geyer et al., 2017) and in drug design (Jiang et al., 2019). The most widely used strategy in LC‐MS‐based proteomics is bottom‐up proteomics (Zhang et al., 2013), in which proteins are first proteolytically digested into peptides, which are usually separated by reversed‐phase high‐performance liquid chromatography (RP‐HPLC). The masses of the peptides are determined at the MS1 level while masses of the corresponding fragment ions are obtained at the MS2 level after fragmentation in a collision cell. By matching the precursor ions measured in MS1 and the fragment ions in MS2 with the theoretical masses generated from in silico digestion of the corresponding protein sequence, the peptides in the sample can be identified and this information is used to assign peptides to the corresponding proteins to arrive at their identification and quantification. A less widely used strategy is top‐down proteomics, which stands for the direct analysis of intact proteins by mass spectrometry. For details about this approach, we refer readers to reviews by Kelleher and coworkers (Catherman et al., 2014; Toby et al., 2016).

As proteomics research progressed, it has become increasingly clear that quantitative information is critical to relate proteomics data to actionable outcomes like the design of drugs or the development of biomarker‐based diagnostic assays. Accurate quantification of the dynamically changing protein composition is the basis for understanding the functioning of biological systems. Based on whether isotope labeling is used or not, existing mass spectrometry‐based proteome‐wide quantitative methods can be classified into (I) label‐free proteomics (Asara et al., 2008; Bondarenko et al., 2002; Christin et al., 2011) and (II) label‐based proteomics (Gygi et al., 1999; Ross et al., 2004; Thompson et al., 2003), also named multiplexed quantitative proteomics. While this review focuses on label‐based proteomics and on chemical labeling in particular, we give a brief introduction of label‐free proteomics in comparison.

### 1.1 Label‐free proteomics

Representative features of label‐free proteomics are discussed below and readers are referred to comprehensive reviews for further details (Bantscheff et al., 2007; Neilson et al., 2011; Zhu et al., 2010). In view of the fact that the label‐free approach does not require derivatization with isotope labels and can be implemented on any type of mass spectrometer, it is widely used with good results due to improvements in instrument reproducibility and advanced data acquisition and data processing schemes (Cox et al., 2014; Cox & Mann, 2008; Röst et al., 2016; Suits et al., 2011). However, label‐free proteomics has inherent limitations in throughput, as it only analyzes one sample per LC‐MS run, and in precision, as it does not correct for analytical variability. Relative quantitation is achieved, for example, by comparing the peak area of precursor ions at the MS1 level or by counting the number of MS2 spectra per peptide (Liu et al., 2004). It is therefore prone to be affected by retention time shifts, changes of ionization efficiency and variations in sample loss during work‐up from injection to injection. To avoid or alleviate these limitations, simultaneous analysis of multiple samples is a good remedy but it requires approaches that allow to discriminate between differentially labeled peptides containing the same sequence in the mass analyzer, while minimally affecting their physicochemical properties to minimize shifts in retention times or variable recoveries during sample work‐up. Hence, stable isotope labeling is an ideal choice as isotopes have almost identical physicochemical properties but distinct masses.

### 1.2 Label‐based proteomics

The technique of multiplexing in proteomics was first introduced in 1999, with the isotope‐coded affinity tag (ICAT) (Colangelo & Williams, 2006; Gygi et al., 1999; Yi et al., 2005) and the ^15^N metabolic labeling method (Oda et al., 1999). After that, a series of other multiplexed quantification approaches have been published, including stable isotope labeling by amino acids in cell culture (SILAC) (Jiang & English, 2002; Ong et al., 2002; Zhu et al., 2002), isobaric tags for relative and absolute quantitation (iTRAQ) (Ow et al., 2008; Ross et al., 2004), tandem mass tags (TMT) (Dayon et al., 2008; Thompson et al., 2003) and isobaric peptide termini labeling (IPTL) (Jiang et al., 2018; Koehler et al., 2009, 2011, 2013; Nie et al., 2011; Zhang et al., 2016). All these approaches exploit different isotope labels to derivatize peptides or proteins that are derived from different samples. Most approaches involve derivatization of proteolytic peptides rather than intact proteins, because every proteolytic peptide contains at least one suitable labeling site, namely a primary amine group, thus permitting the labeling of all peptides in a sample using the same strategy. Since the differentially labeled peptides can be resolved by mass spectrometry and the labeled samples are pooled before LC‐MS (Chahrour et al., 2015; Rauniyar & Yates, 2014; Zhou et al., 2014), simultaneous analysis of multiple samples can be achieved, which circumvents signal variation due to, among others, variable sample loss during work‐up and changing ionization efficiency between injections (Bantscheff et al., 2007; Krijgsveld et al., 2003; Sonnett et al., 2018). Multiplexed approaches are expected to afford better quantification precision and have a higher throughput thus reducing the overall analysis time per sample. The commonly used heavy isotopes are ^13^C, ^15^N, ^18^O, and ^2^H, with ^2^H being less popular due to the potential risk of varying LC retention times of the same peptide modified with a different number of deuterium atoms (Boutilier et al., 2012; Di Palma et al., 2011; Koehler et al., 2015; Zhang et al., 2002), which is caused by the differential interaction between hydrogen/deuterium and the reversed‐phase stationary phase, since deuterium has a larger mass and smaller amplitude of vibrations than hydrogen (Turowski et al., 2003; Zhang et al., 2001). Hence, in spite of the fact that deuterium‐based labels can extend the multiplexing capacity further, the majority of chemical labeling approaches utilizes ^13^C and ^15^N isotopes.

**Figure 1:**
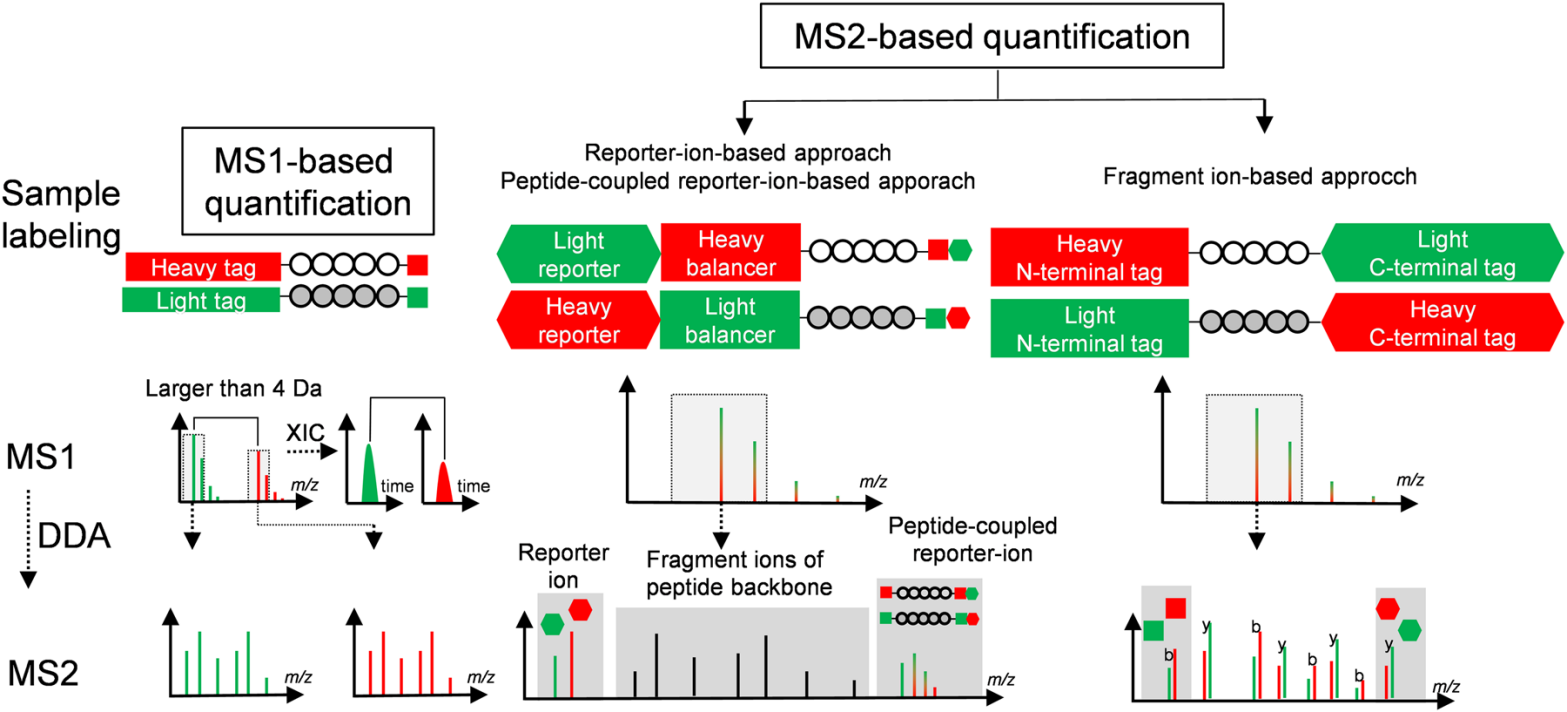
Schematic overview of MS1‐based and MS2‐based quantification [Color figure can be viewed at wileyonlinelibrary.com]

A large number of multiplexed methods have been reported, which can be classified into sub‐categories according to different criteria, as shown in Figure 1: (I) based on the stage at which peptides are quantified, they can be categorized into MS1‐based quantification (e.g., ICAT (Colangelo & Williams, 2006; Gygi et al., 1999; Yi et al., 2005) and mTRAQ (Kang et al., 2010; Mertins et al., 2012)) and MS2‐based quantification (e.g., TMT and iTRAQ); (II) based on whether the same peptides derived from different samples have the same mass after isotope labeling or not, they can be divided into isobaric labeling‐based quantification (TMT, IPTL (Koehler et al., 2009) and EASI tag (Winter et al., 2018)), or isotopic labeling‐based quantification (ICAT and m‐pIDL (Liu et al., 2019)); (III) based on the approach of incorporating the isotopes, they can be classified into chemical labeling‐based quantification (e.g., ICAT and TMT), enzymatic labeling‐based quantification (^18^O labeling (Sakai et al., 2005; Yao et al., 2001)) or metabolic labeling‐based quantification (SILAC (Jiang & English,^2002^; Ong et al., 2002; Zhu et al., 2002)). (IV) based on whether ultra‐high resolution is required or not to distinguish mDa mass differences at the MS1 or MS2 level, they can be subcategorized into tags not requiring Ultrahigh resolution (e.g., ICAT and 4‐plex iTRAQ), tags requiring Ultrahigh resolution at the MS1 level (DiPyrO and mdDiLeu) and tags requiring Ultrahigh resolution at the MS2 level (11‐plex TMT and 21‐plex DiLeu). It is notable that these classifications are based on different criteria (Table 1), which are not mutually exclusive. Although some tags, like 4‐plex iTRAQ and DiLeu, do not have strictly the same precursor masses, they are classified as isobaric, since the mDa differences of precursor masses are not used at the MS1 level to discriminate between different labeling channels. Throughout this review, we will focus on chemical labeling‐based quantitative proteomics approaches, in which proteolytic peptides are chemically labeled with distinct isotope labels and labeled samples are then pooled and analyzed in a single LC‐MS run.

**Table 1:** Classification of chemical isotope labeling approaches based on different criteria

	MS1quantification	MS2quantification	Isotopiclabeling	Isobariclabeling	Ultra‐high resolution at MS1	Ultra‐high resolution at MS2
(c)ICAT	**√**		**√**			
Dimethylation	**√**		**√**			
ICPL	**√**		**√**			
mTRAQ	**√**		**√**			
mTMT	**√**		**√**			
DiPyrO	**√**		**√**		**√**	
mdDiLeu	**√**		**√**		**√**	
iTRAQ^a^		**√**		**√**		
TMT		**√**		**√**		**√**
DiLeu^a^		**√**		**√**		
IBT		**√**		**√**		**√**
DiART		**√**		**√**		**√**
IPTL		**√**		**√**		**√**
m‐pIDL		**√**	**√**			**√**
Ac‐IPG		**√**		**√**		
TMTc(+)		**√**		**√**		
EASI		**√**		**√**		
Ac‐AG		**√**		**√**		

^a^ Although the iTRAQ and DiLeu series labels have mDa differences in precursor masses, they are classified as isobaric since the mDa differences are not revealed at the MS1 level in general use and they are already habitually referred to as isobaric.

### 1.3 Data acquisition strategies

**Figure 2:**
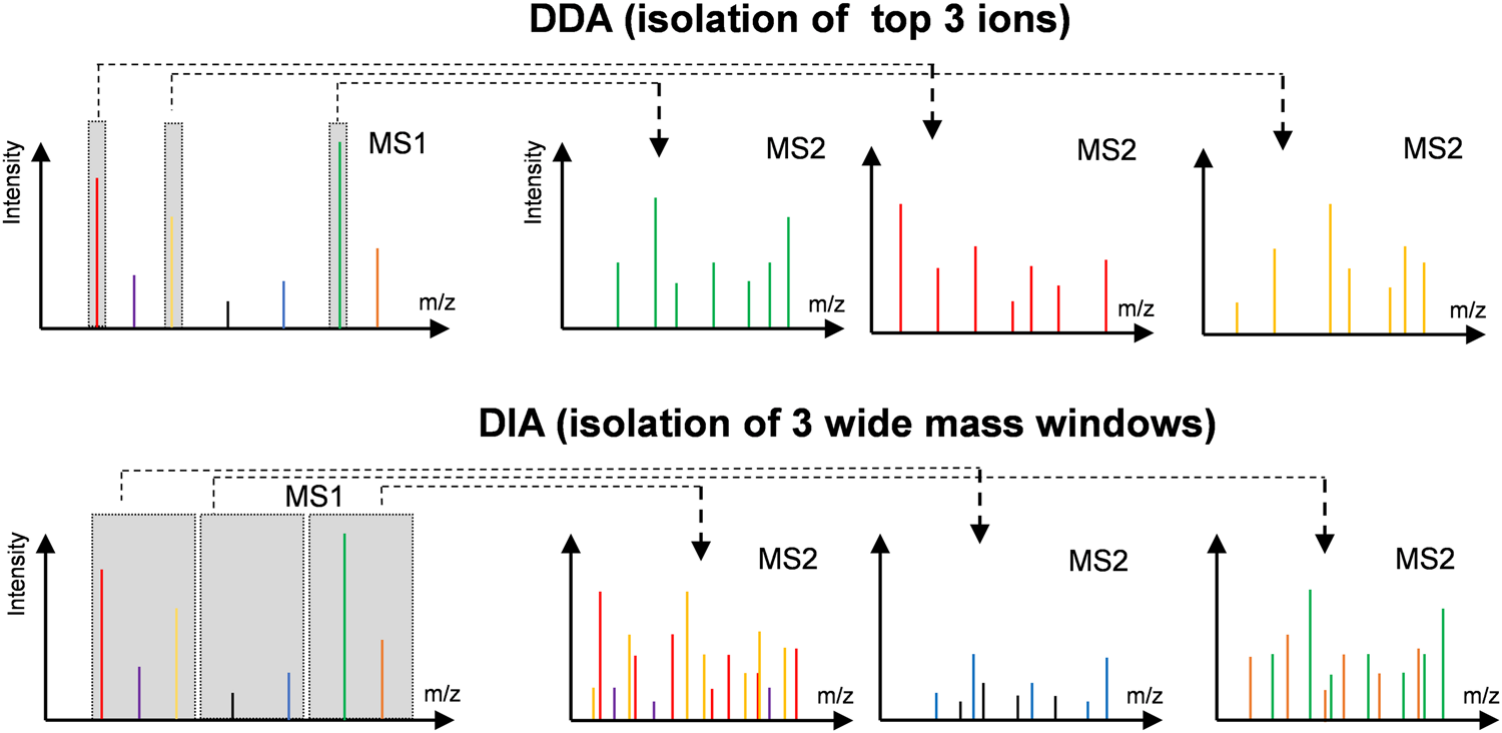
Difference in MS1 isolation windows for the DDA and DIA modes. DDA, data‐dependent acquisition; DIA, data‐independent acquisition [Color figure can be viewed at wileyonlinelibrary.com]

Before discussing specific labeling strategies, it is necessary to introduce the main features of the two main MS2 spectra acquisition modes, namely data‐dependent acquisition (DDA) and data‐independent acquisition (DIA), as they are closely linked to the advantages and disadvantages of multiplexed quantification approaches. In most multiplexed methods, collecting MS2 spectra is performed in DDA mode, in which a certain number of precursor ions is successively, but individually, selected for fragmentation during an LC‐MS run in order of decreasing intensity. The resulting fragments are collected in discrete MS2 spectra thus providing a direct link between precursor ion and its fragments (Stahl et al., 1996). DDA therefore has a bias toward high‐abundance species and limits the detection of low‐abundance peptides and ultimately proteins (Figure 2). The number of peaks that can be isolated for fragmentation increases with the scanning rate of the mass spectrometer, but there are usually more peaks in the MS1 spectra than the instrument can isolate in the timeframe of the analysis, usually the width of an LC peak, resulting in minor peaks being ignored. This problem increases with increasing complexity of the samples and is further related to the resolution of the chromatographic separation leading to the fact that highly complex samples must often be fractionated before the final LC‐MS analysis, which increases the analysis time per sample. In addition, the stochastic nature of precursor ion selection in the DDA mode diminishes the run‐to‐run reproducibility often leading to the so‐called “missing value problem,” which means that a given peptide may not be detected (fragmented) in every LC‐MS run (Hu et al., 2016; Venable et al., 2004). This creates a problem for comparative data analysis. Various methods have been developed to arrive at a more comprehensive sampling of precursor ions in DDA (Pelletier et al., 2020; Schweppe et al., 2020). Dynamic exclusion is a commonly used strategy to prevent a peptide from triggering multiple MS2 events during the timeframe of an eluting chromatographic peak, as this dedicates time to a potentially already identified peptide while others escape analysis. Despite these limitations, DDA is still the most widely used data acquisition strategy in current discovery proteomics, because of well‐developed data acquisition and data processing workflows that can be implemented on almost any MS instrument.

In contrast to DDA, isolation of precursors for fragmentation in DIA does not rely on peak intensities in MS1. Instead, all precursors present within a predefined isolation window will be fragmented and analyzed together, as shown in Figure 2. DIA largely addresses the problem of missing values (Gillet et al., 2012), however, at the price that the direct link between precursor and fragment ions is lost. MS2 spectra of DIA are inherently more complex, since fragment ions derived from multiple, co‐isolated precursor ions are present in the same MS2 spectrum, which makes analyzing DIA data more challenging than DDA data. Since the Sequential Windowed Acquisition of all Theoretical fragment ion Mass Spectra (SWATH‐MS) (Gillet et al., 2012) was proposed, DIA has made remarkable advances in recent years with the development of sophisticated data processing algorithms (Bern et al., 2010; Collins et al., 2013; Gillet et al., 2012; Rardin et al., 2015). It is also notable that both the MS1 and MS2 data can be used for quantitation in DIA mode (Huang et al., 2020; Rardin et al., 2015). While the application of stable isotope labeling is widely used in DDA mode, it is still in its infancy when it comes to DIA. At the end of this review, we will therefore highlight current developments, advantages and challenges of applying stable isotope labeling in DIA mode.

## 2 MS1‐BASED QUANTIFICATION

MS1‐based quantitative approaches are based on introducing distinct mass additions to peptides by isotopic labeling, so that the same peptides derived from different samples have different masses. Quantification is achieved by comparing the peak areas or intensities of peptide ions for each labeling channel at the MS1 level (Gygi et al., 1999; Hsu et al., 2003; Schmidt, 2005). Subsequently, the quantitative information at the peptide level needs to be transferred to the protein level (Nesvizhskii et al., 2003), which is a common challenge for DDA and DIA. Usually, a mass shift of at least 4 Da is incorporated into the isotopic tags to avoid overlap between the isotope envelopes of light‐ or heavy‐labeled samples (Boersema et al., 2008). The overlap resulting from smaller mass shifts (<4 Da) renders relative quantification between differentially labeled peptide pairs/clusters more difficult since an additional deconvolution step is required. This is particularly challenging for deuterium containing labels, which induce a slight shift in retention time (Choi et al., 2020).

The distinct isotopes for MS1‐based quantification can be incorporated into samples by three approaches: (1) chemical labeling (e.g., ICAT (Gygi et al., 1999) and mTMT (Paulo & Gygi, 2019)) with synthetic tags targeted at specific reactive groups (amine or thiol); (2) enzymatic labeling (Sakai et al., 2005; Yao et al., 2001), where proteins are enzymatically digested in the presence of ^18^O labeled water and two ^18^O atoms are incorporated into the newly formed C‐terminal carboxyl groups of digested peptides (Miyagi & Rao, 2007; Zhao et al., 2010); (3) metabolic labeling, such as SILAC (Jiang & English, 2002; Ong et al., 2002; Zhu et al., 2002), where distinct isotope‐labeled amino acids (e.g., Lys and Arg) are fed to cell cultures and metabolically incorporated into newly synthesized proteins. In the following discussion, we will focus on chemical labeling approaches. For in‐depth discussions about enzymatic labeling‐based and metabolic labeling‐based quantification, we refer readers to other comprehensive reviews (Chahrour et al., 2015; Liu et al., 2020).

### 2.1 ICAT and cICAT tags

**Figure 3:**
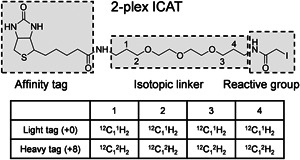
Structure design and isotope distribution of 2‐plex ICAT. ICAT, isotope‐coded affinity tag

The first reported chemical isotope labeling tag is the ICAT (Gygi et al., 1999; Han et al., 2001; Smolka et al., 2001) that consists of three functional parts, as shown in Figure 3: (1) a biotin moiety, which can be used to enrich the ICAT‐labeled peptides from a digest mixture via (strept) avidin affinity chromatography; (2) an isotopic linker, which contains either eight ^2^H or ^1^H atoms to differentially label peptides from different samples; (3) an iodoacetamide group, which specifically reacts with the sulfhydryl group at the side chain of cysteine residues. Compared to label‐free methods, the main improvement of ICAT was the ability to simultaneously quantify two samples in a single LC‐MS run based on a subset of peptides containing at least one cysteine, which reduces sample complexity while preserving the identification coverage, since most proteins contain at least one cysteine residue despite the fact that cysteine is a rather rare amino acid (Yi et al., 2005). Although the merits of ICAT were clear, several limitations quickly came to light (Hansen et al., 2003): first, the employed ^2^H atoms in the linker alter the retention time in comparison to the ^1^H‐containing peptide. Second, fragmentation occurs at the biotin moiety, adversely impacting peptide identification due to unassignable fragment ions. Third, due the large mass of ICAT, labeled peptides may shift out of the optimum *m*/*z* range for detection, especially when more than one tag is incorporated in a peptide. In spite of these limitations, ICAT is widely considered as the pioneer of isotope labeling‐based quantitative proteomics based on chemical labeling. Subsequently, a cleavable ICAT (cICAT) was proposed (Hansen et al., 2003; Li et al., 2003; Oda et al., 2003; Parker et al., 2004; Yu et al., 2004), that allows removal of the biotin moiety before LC‐MS at acidic pH thus reducing problems with peptide fragmentation. In addition, replacing ^2^H with ^13^C in the linker ensures the coelution of differentially labeled peptides during reversed‐phase chromatography. However, the specificity of cICAT for cysteine remains a double‐edged sword. It reduces the complexity of the proteome sample to allow a more detailed analysis of the subset of peptides containing cysteine, while missing all information about peptides containing no cysteine.

### 2.2 Dimethylation

**Figure 4:**
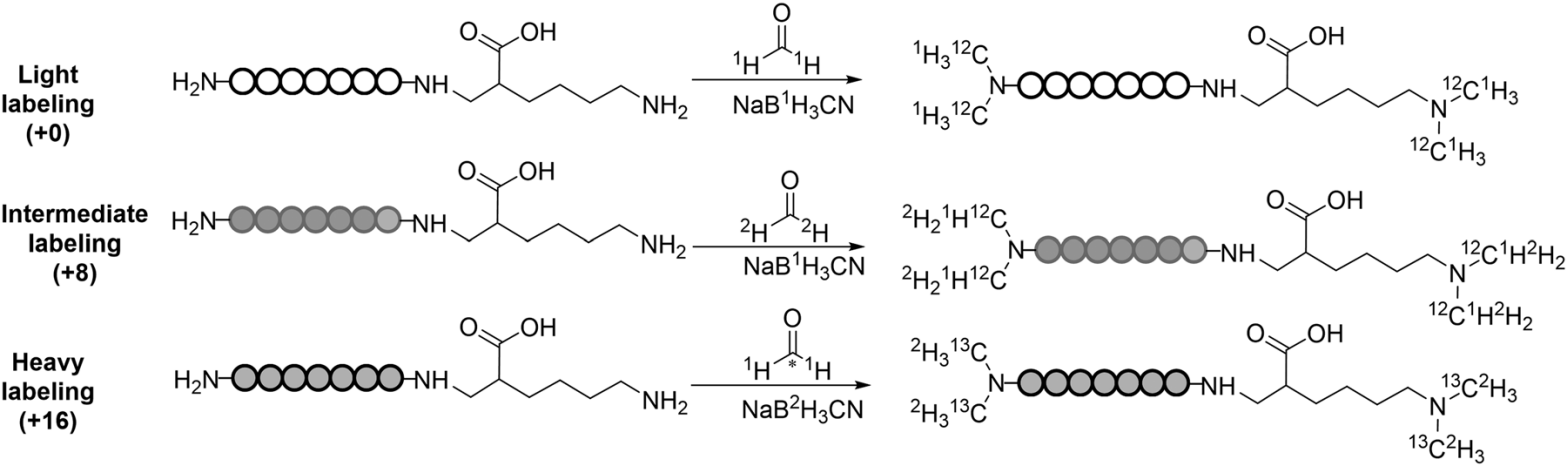
Triplex dimethylation of a peptide with a C‐terminal Lys residue. ^13^C in formaldehyde is marked with an asterisk

Dimethylation, a simple and cost‐effective chemical labeling strategy, was first proposed in 2003 (Hsu et al., 2003). It is based on reductive amination chemistry, where the N‐terminal amine group of a peptide/protein and the amine group at the side chain of Lys residues are reacted with formaldehyde, which is available as ^12^C^1^H_2_O, ^13^C^1^H_2_O, ^12^C^2^H_2_O, and ^13^C^2^H_2_O, followed by reduction with sodium cyanoborohydride, which is available as NaB^1^H_3_CN and NaB^2^H_3_CN. As shown in Figure 4, with combinations of different isotopic forms, dimethylation can achieve duplex or triplex labeling. By applying ^12^C^1^H_2_O with NaB^1^H_3_CN, ^12^C^2^H_2_O with NaB^1^H_3_CN and ^13^C^2^H_2_O with NaB^2^H_3_CN, triplex labeling of “light, intermediate and heavy” can be achieved with a mass shift of 4 Da per derivatized site (Boersema et al., 2008, 2009). Instead of tagging at a rare amino acid, like Cys in ICAT, dimethylation achieves isotopic labeling at the amine group which exists in almost all proteolytic peptides enabling identification and quantification of proteins based on multiple peptides at the cost of a more complex peptide mixture. Dimethylation increases peptide ionization efficiency, as the resulting tertiary amine is easier ionized in electrospray, and improves completeness of the b‐ion series (Fu & Li, 2005). Another interesting observation is that peptides that are differentially dimethylated with different numbers of deuterium atoms show a smaller retention time shift compared to that observed in ICAT (Hansen et al., 2003). Presumably, deuterium atoms on a hydrophilic trimethylamine group have less interactions with the reversed‐phase stationary phase than those in the ethyleneglycol linkers of ICAT tags (Boersema et al., 2008; Hsu et al., 2003; Zhang et al., 2002). Selective N‐terminal dimethylation is an adaptation of dimethylation (Qin et al., 2012), that exploits the pK_a_ difference between the N‐terminal amine group and the amine group at the side chain of Lys residues. Diethylation, using the same labeling principle as dimethylation, but replacing formaldehyde with acetaldehyde thus offering a higher multiplexing capacity, was recently reported (Choi et al., 2020; Jung et al., 2019). By comparison with the deuterium‐based triplex‐dimethylation, the ^13^C‐based triplex‐diethylation method exhibited better quantitative accuracy and precision (Jung et al., 2019), mainly because of the absence of the deuterium effect on retention time.

### 2.3 Isotope‐coded protein label (ICPL), mTRAQ, and mTMT tags

**Figure 5:**
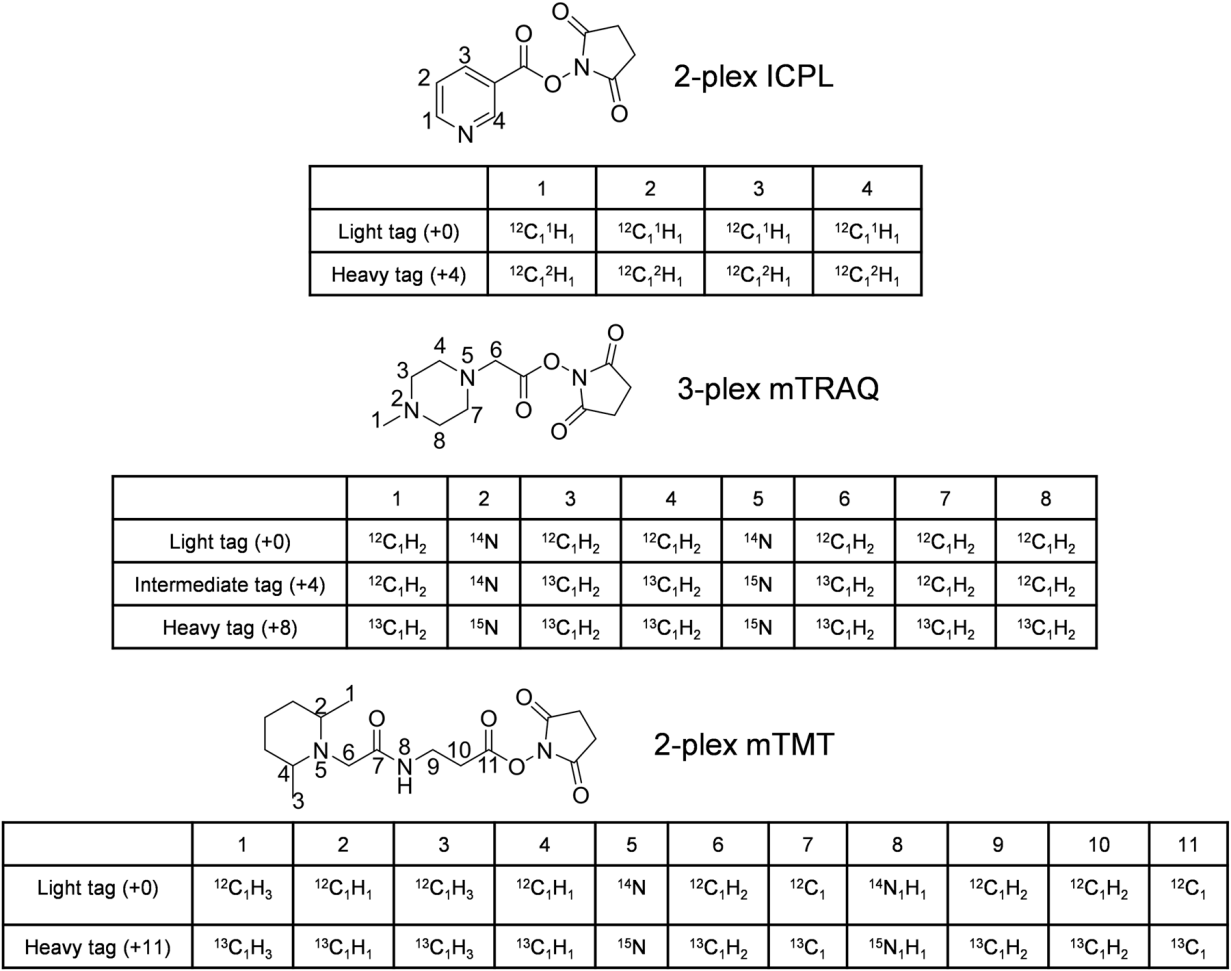
The structure and isotope distribution of 2‐plex ICPL, 3‐plex mTRAQ and 2‐plex mTMT tags

The ICPL was first introduced in 2005 (Schmidt, 2005). The tag contains a pyridine ring that ionizes well and an *N*‐hydroxysuccinimide (NHS) ester to permit the efficient labeling of N‐terminal ɑ‐amine groups and the epsilon‐amine group of Lys (Com et al., 2012; Yu et al., 2017). In the original version of the ICPL method (Figure 5), the ^1^H atoms on the pyridine ring of the “light tag (+0)” were substituted with ^2^H to generate the “heavy tag (+4),” allowing duplex experiments. To avoid retention time shifts due to the deuterium effect, another version of ICPL with^13^C was developed (Yu et al., 2017). In the original ICPL work‐flow, samples were labeled at the protein level, avoiding ratio deviations caused by different digestion efficiency and sample loss during the digestion process. However, this strategy is limited by the fact that only 60%–70% of identified proteins can subsequently be quantified (Chahrour et al., 2015; Leroy et al., 2010), presumably because not all identified peptides contain the ICPL tag, as not all primary amines are equally accessibly in intact proteins. It is by far most common to conduct labeling after digestion, since every proteolytic peptide contains at least one amine group at the N‐terminus. As a result, every peptide conveys quantitative information, which significantly increased the proportion of quantified proteins to 98% for ICPL (Leroy et al., 2010).

The mTRAQ label was first used in discovery proteomics for global relative quantification in 2010 (Kang et al., 2010). As shown in Figure 5, mTRAQ has a tertiary amine in the structure for efficient ionization and is available in three isotope forms (+0, +4, and +8) resulting in mass shifts of 140, 144, and 148 Da per derivatized site, respectively. The mTMT tag, proposed in 2019 (Paulo & Gygi, 2019), has the same chemical structure as the isobaric TMT tag that will be discussed later. In the original report, the TMTzero (TMT0) and “super heavy” TMT (shTMT) tags were used to differentially label two samples with a mass difference of 11 Da (Figure 5). Both mTRAQ and mTMT have an amine reactive NHS ester, which enables the efficient labeling of primary amine groups in peptides. Both tags use only ^15^N and ^13^C atoms in the heavy labels.

### 2.4 Neutron‐encoded isotopic labeling

**Figure 6:**
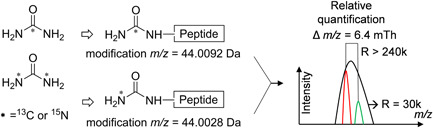
The concept of quantification based on neutron encoding. ^13^C and ^15^N are marked with an asterisk. The 6.4 mDa mass difference can be differentiated at resolutions higher than 240k in the *m*/*z* 100–1000 range. The peak intensities represent relative quantification [Color figure can be viewed at wileyonlinelibrary.com]

**Figure 7:**
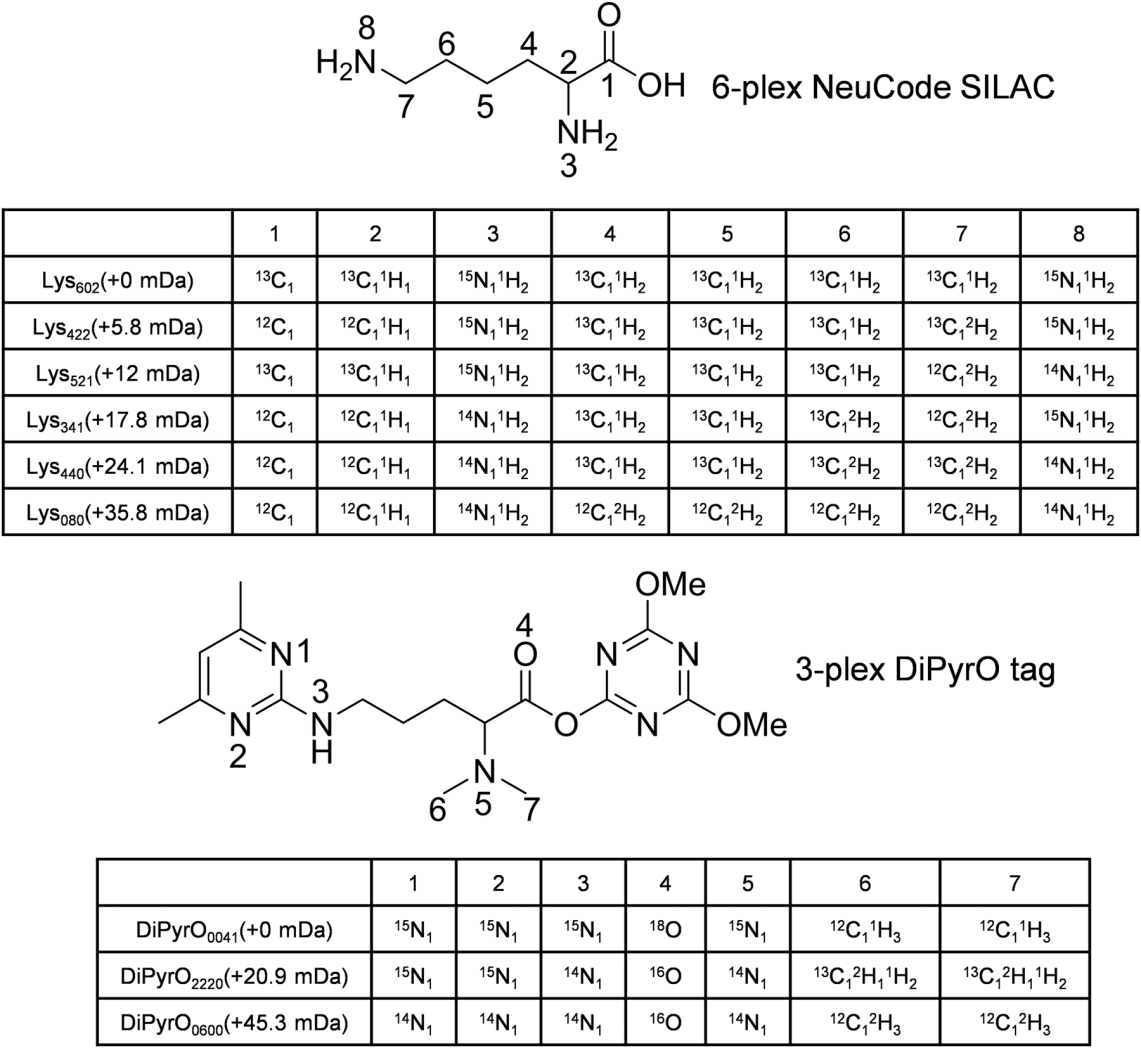
Structure and isotope distribution of 6‐plex NeuCode SILAC lysine and the 3‐plex DiPyrO tag

Neutron encoding is used for quantifying peptides based on the mDa mass difference obtained by incorporating either ^2^H, ^13^C, or ^15^N isotopes (see in Figure 6), which extends the multiplexing possibilities of both chemical and metabolic labeling strategies in MS1‐ and MS2‐based quantitative proteomics (Hebert, Merrill, Bailey, et al., 2013; McAlister et al., 2012). MS1‐based quantification approaches generally use a mass spacing of at least 4 Da between labeling channels to avoid overlap between the isotope envelopes of differentially labeled peptides. However, this spacing restricts the number of isotopes that can be added and thus constrains the multiplexing capacity. Inspired by increasing the multiplexing capacity with the mDa difference obtained by neutron encoding in the isobaric label 8‐plex TMT (see Section 3.1.1) (McAlister et al., 2012; Werner et al., 2012), Hebert et al. first introduced neutron encoding as an MS1‐based quantification approach in 2013 for metabolic labeling with amino acids in cell culture (Hebert, Merrill, Bailey, et al., 2013). In that report, they proposed NeuCode SILAC that achieved multiplexed labeling by using two kinds of Lys, one containing six ^13^C and two ^15^N atoms (“heavy 1,” +8.0142 Da) and another containing eight ^2^H atoms (“heavy 2,” +8.0502 Da) for the metabolic labeling of yeast proteins. Compared to unlabeled Lys, both isotopically labeled Lys involve a ~8 Da mass increase but the small mass difference of 36 mDa between them can be distinguished at the MS1 level at a resolution above 200k, which is achievable in Fourier Transform Ion Cyclotron Resonance (FT‐ICR) and recent Orbitrap mass analyzers. A scanning scheme including three sections was applied: (1) a high‐resolution MS1 scan, which is used for quantification; (2) a modest‐resolution MS1 scan, in which the mDa difference is not detectable and from which the precursors are selected for fragmentation; and (3) multiple MS2 scans with modest‐resolution to generate data for peptide identification. The most attractive feature of this approach is that precursor pairs of the same peptide are isolated and fragmented together to produce indistinguishable MS2 spectra at a low resolution. In this way, the complexity of the MS1 spectra does not affect precursor ion selection and light and heavy forms of the same peptide are selected in the same window. This benefits the overall cycle time and increases the intensity of the overlapping fragment ions produced from both channels facilitating identification. Two factors determine the multiplexing capacity of the neutron encoding‐based approach: (1) the number of available isotopologues; and (2) the attainable resolution. The more isotopologues are considered, the higher the required resolution. By adding four new Lys isotopologues (K_#13C#2H#15N_: K_422_, K_521_, K_341_, and K_440_) as shown in Figure 7, a 6‐plex neutron‐encoded SILAC experiment was performed (Merrill et al., 2014; Overmyer et al., 2018).

The NeuCode approach has also been applied to chemical labeling MS1‐based quantitation, using ^13^C and ^15^N in the 4‐plex amine reactive acetyl‐Arg‐(acetyl)Lys‐Gly‐NHS tag having a 12.6 mDa mass difference between channels (Hebert, Merrill, Stefely, et al., 2013). However, the performance of the tag was suboptimal with a low number of identified peptides presumably caused by (1) the bulkiness of the tag leading to fragmentation of the tag itself generating sequence‐uninformative fragment ions; and (2) sequestering protons on the Arg residue in the tag restricting fragmentation of the peptide backbone (Hebert, Merrill, Stefely, et al., 2013). To avoid these problems, various neutron‐encoded chemical tags, that are more compact and facilitate peptide fragmentation, have been reported, such as neutron‐encoded carbamylation (Ulbrich et al., 2014) (Figure 6), mdDiLeu (Hao et al., 2017; Zhong et al., 2019) and DiPyrO (Frost et al., 2017). As asserted in the work on DiPyrO (Figure 7), deuterium is often needed to construct tags for a higher multiplexing capacity while ensuring the compactness of tag, which may affect the chromatographic retention of the same peptide labeled with a different number of deuterium atoms. Neutron encoding‐based quantification can be improved in two ways: (1) by working with amine reactive tags, digestion with LysC instead of trypsin permits all peptides to accept two neutron‐encoded tags, which doubles the mass difference between channels thus effectively reducing the requirements in terms of resolving power; (2) by combining the strategy of mass differences between isotopologues of the same element and of different elements to improve multiplexing capacity. For example, the acetyl‐Arg‐(acetyl)Lys‐Gly‐NHS tag allowed 12‐plex labeling by triplicating the NeuCode‐based 4‐plex labeling (Hebert, Merrill, Stefely, et al., 2013): +0 Da (+0, +12.6, +25.2, +37.8 mDa), +4 Da (+0, +12.6, +25.2, +37.8 mDa), +8 Da (+0, +12.6, +25.2, +37.8 mDa). Overall, compared to the conventional MS1‐based quantification approaches, the neutron‐encoded MS1 approaches have advantages in terms of sampling depth, since redundant MS2 spectra of differentially labeled peptides are avoided, and in terms of multiplexing capability. However, they require very high resolution at the MS1 level, which increases the cycle time in Fourier Transform‐based mass analyzers.

### 2.5 Summary of MS1‐based quantification

To conclude, all MS1‐based quantification approaches have the common strength of simultaneously analyzing multiple samples in a single LC‐MS run. Therefore, relative quantitation between samples is performed in the same run rather than in different runs, which improves quantification precision. However, multiplexing capacity is usually limited for two reasons. First, isotopic labeling in MS1‐based quantification approaches multiplies the complexity of MS1 spectra with the number of labeled samples, since the same peptides derived from different samples have distinct masses. The increase in MS1 complexity is a disadvantage when using DDA for data acquisition, since only a limited number of precursor ions can be selected for fragmentation within the timeframe of a chromatographic peak, resulting in more peaks being neglected for complex samples. Moreover, abundant peptides appearing as multiplets may be triggered multiple times, which leads to redundant MS2 spectra of the same peptide and consumes precious time that could be used to fragment peptides from lower abundant proteins (Boersema et al., 2008). The second limitation with respect to increasing the multiplexing capacity lies in the requirement for more atoms to convey isotopes and the mass shift of at least 4 Da between labeling channels to prevent overlap of isotope envelopes. For both reasons, a large isotopic tag is required, which may affect the fragmentation properties negatively and result in a decline in identification rate (Ow et al., 2009; Pappireddi et al., 2019; Pichler et al., 2010). The neutron encoding approaches improve the multiplexing capacity without interfering with precursor ion selection. However, its feasibility is highly resolution‐dependent (>200k) and requires state‐of‐the‐art FT‐ICR or Orbitrap mass analyzers. Besides, being restricted by the compactness of the tag, and hence the limited number of locations that can be labeled with ^13^C/^15^N, as well as the limited commercial availability of the tags, neutron encoding‐based MS1 tags are currently not in widespread use. Besides, we refer readers to other interesting applications of MS1 tags in functional proteomics studies (Weerapana et al., 2010).

## 3 MS2‐BASED QUANTIFICATION

As discussed above, MS1‐based quantification has a multiplexing capacity that is generally limited to triplex labeling. However, exploring biological questions often requires quantitative comparison of proteomes across multiple conditions at different time points resulting in more than three samples. MS2‐based quantification approaches readily allow for higher multiplexing capacity without most of the drawbacks of MS1‐based quantification methods.

MS2‐based quantification generally relies on labeling of the same peptides derived from different samples with different isobaric tags. As a result, differentially labeled peptides have identical masses, which not only avoids an increase in MS1 spectrum complexity but also increases signal intensity of peptide ions by combining the signals from each channel. Isobaric labeling further reduces the risk of acquiring redundant MS2 spectra from the same peptide. This makes isobaric labeling highly compatible with the DDA acquisition mode, where only a certain number of precursor ions can be isolated for fragmentation within a given time window. Except for the fragment ion‐based quantification variant, isobarically labeled peptides of different samples produce the same sequence‐derived fragment ions of the peptide backbone, which affords a better signal‐to‐noise (S/N) ratio and thus improves peptide identification.

Upon fragmentation, isobarically labeled peptides release unique quantification ions, often called reporter ions, for each labeling channel. The relative peak intensities of these ions are proportional to the corresponding peptide levels (Thompson et al., 2003). By comparing the intensities of the quantification ions, the relative quantitative information can be retrieved from the MS2 spectra which generally have a better S/N ratio than MS1 spectra (Venable et al., 2004; Zhang & Neubert, 2006). Deuterium‐based labeling is avoided in most isobaric tags, since coelution of differentially labeled peptides containing the same sequence is critical for quantification accuracy. Based on the type of quantification ion, the current isobaric labeling methods fall into three general categories: (1) reporter ion‐based quantification (e.g., TMT (Dayon et al., 2008; Thompson et al., 2003), iTRAQ (Ross et al., 2004)), (2) peptide fragment ion‐based quantification (e.g., IPTL (Koehler et al., 2009), Ac‐IPG (Tian et al., 2020a)) and (3) peptide‐coupled reporter ion‐based quantification (e.g., TMTc (Sonnett et al., 2018,Wühr et al., 2012), EASI (Winter et al., 2018) and Ac‐AG (Tian et al., 2020b)).

### 3.1 Reporter ion‐based quantification

As reviewed by Arul and Robinson (2019), reporter ion‐based quantification is the most widely used isobaric labeling‐based strategy, and includes TMT, iTRAQ and DiLeu. The structural design of reporter‐ion‐based isobaric tags consists of three components: (1) a reporter‐ion moiety, containing distinct masses for different labeling channels thus representing the quantitative information of the constituent peptides in MS2, (2) a balancer group, containing complementary isotopes to the reporter‐ion to ensure the same overall mass of the isobaric tags, and (3) a reactive group, targeting a specific functional group on the peptide.

#### 3.1.1 TMT and iTRAQ tags

**Figure 8:**
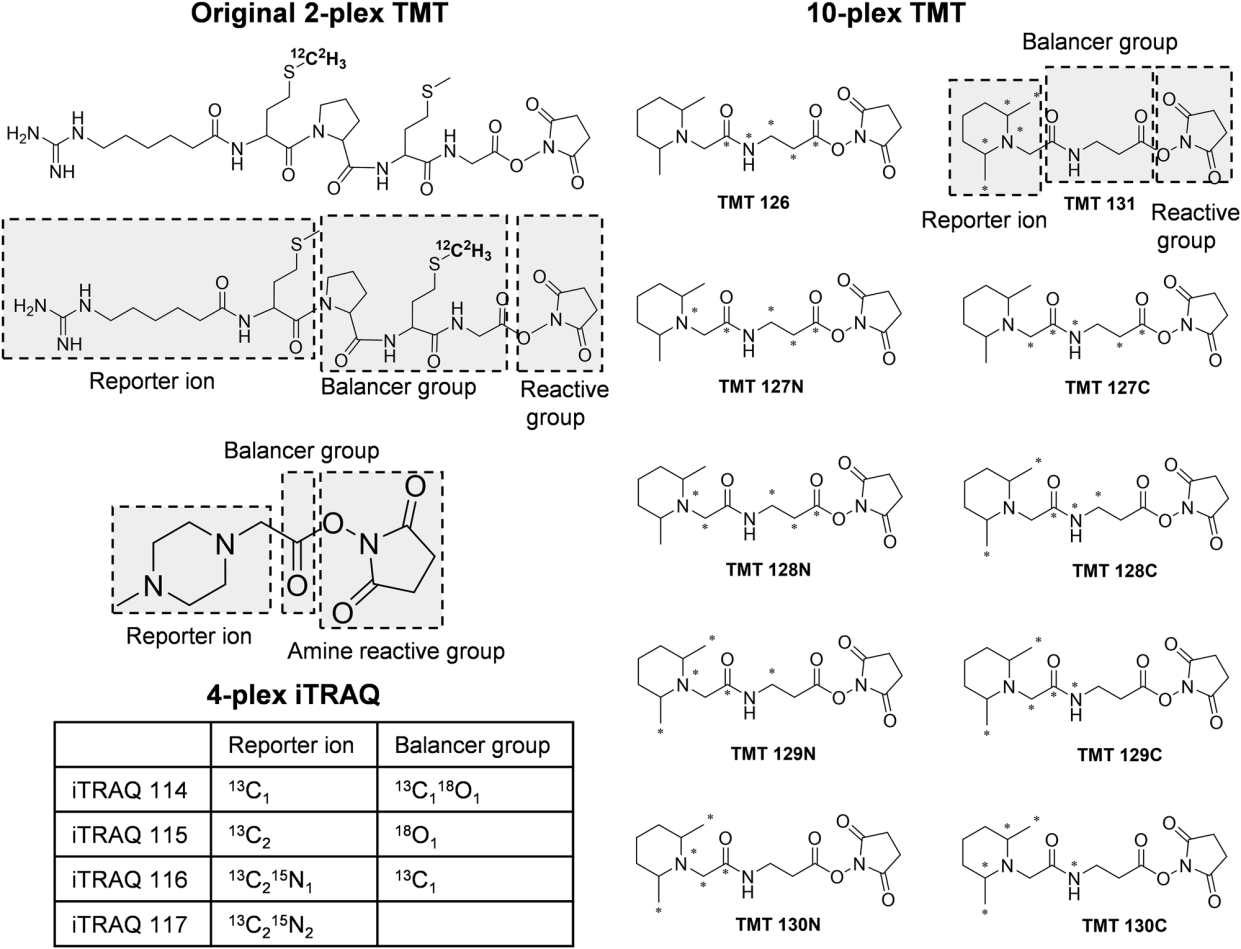
The concept of isobaric tags, with reporter ion, balancer and amine reactive groups. Fragmentation occurs between the balancer and the reporter ion, which carries the quantitative information. The isotope distribution of the original 2‐plex TMT, the 4‐plex iTRAQ and the 10‐plex TMT (with neutron encoding) are shown as representative examples. For 10‐plex TMT ^13^C and ^15^N are marked with an asterisk

**Figure 9:**
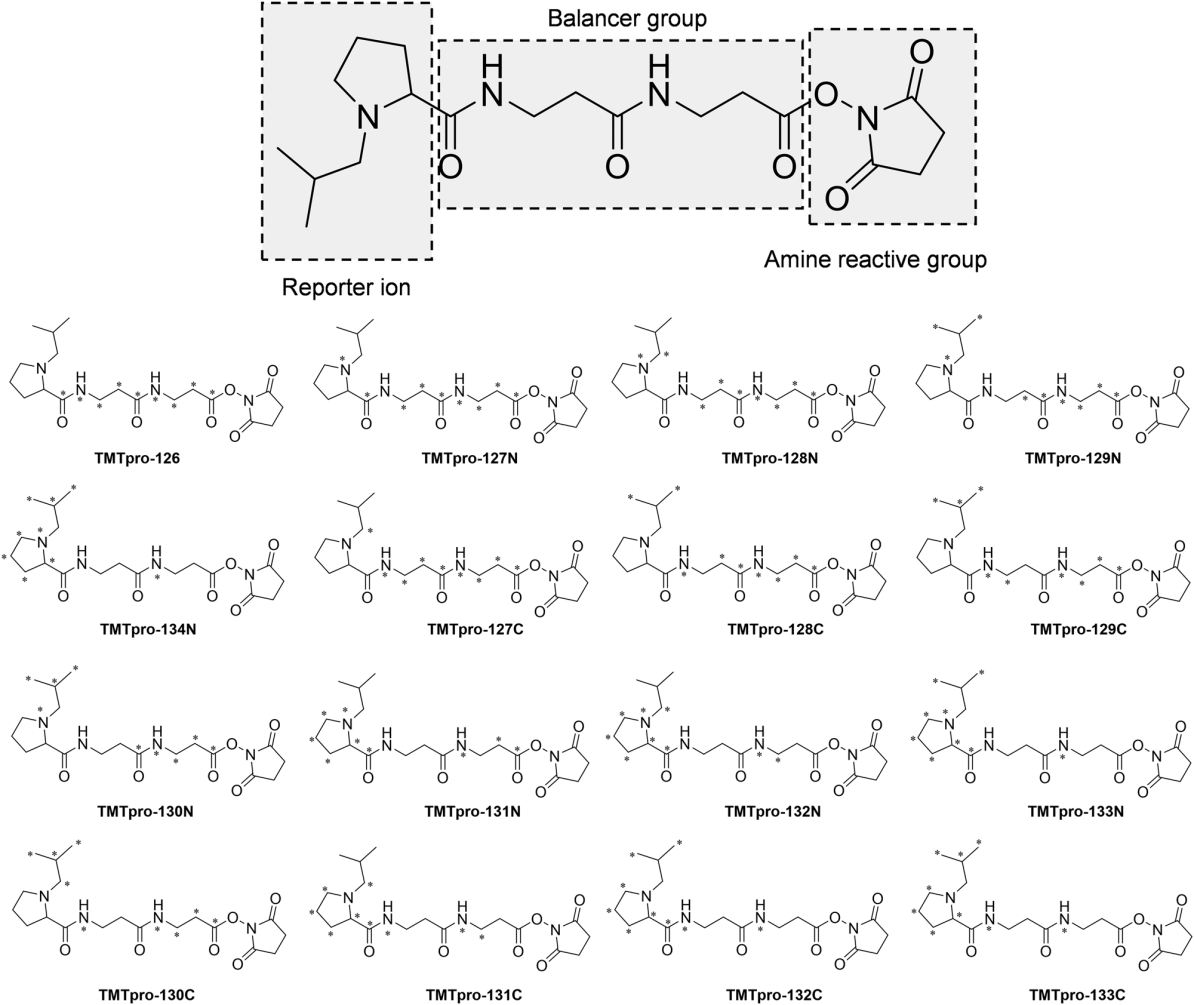
Structure and isotope distribution of the 16‐plex TMTpro tag. ^13^C and ^15^N are marked with an asterisk

The concept of isobaric labeling was first proposed in 2003 with the original version of the 2‐plex TMT (Thompson et al., 2003). It represented the next breakthrough in quantitative proteomics after ICAT. In the original paper, two structures, 6‐guanidinohexanoic acid‐Met‐Met‐Gly‐NHS and 6‐guanidinohexanoic acid‐Met‐Pro‐Met‐Gly‐NHS (Figure 8), were proposed. Fragmentation at the N‐terminal side of the proline residue leads to formation of the reporter ion, permitting the simultaneous acquisition of fragment ions of the peptide backbone and reporter ions. In 2008, an upgraded 6‐plex TMT design was reported (Dayon et al., 2008). Compared to the first‐generation TMT (Thompson et al., 2003), the structure of 6‐plex TMT is more compact and optimized to facilitate the formation of the reporter ion. It was further extended to 10‐plex labeling by neutron encoding (McAlister et al., 2012; Werner et al., 2012). As in all recent labeling methods, only ^13^C and ^15^N isotopes are used while deuterium is avoided (Figure 8). The low‐mass reporter ions with a minimal mass shift of 6.3 mDa can be differentiated by mass analyzers at a resolution of 50k (McAlister et al., 2012) at *m*/*z* 100 which is lower than the resolution of 200k as required for neutron‐encoded MS1‐based quantification. By substituting every ^12^C with ^13^C and every ^14^N with ^15^N, 18‐plex can be achieved based on the chemical skeleton of the 6‐plex TMT (McAlister et al., 2012; Thompson et al., 2019). However, presumably due to restrictions of chemical synthesis and reagent cost, 11‐plex is currently the highest multiplexing capacity for this TMT design. Driven by the demand for even higher multiplexing capacity and presumably lower synthesis cost, a 16‐plex proline‐based isobaric Tandem Mass Tag (TMTpro) was recently introduced, which uses labeled proline as reporter ion rather than dimethylpiperidine and incorporates two β‐alanine residues as the balancer group (Li et al., 2020; Thompson et al., 2019) (Figure 9). In the TMTpro tag, incorporation of 9 heavy isotopes (^13^C and ^15^N) results in 9 tags with a ~1 Da mass shift and applying neutron encoding permits 7 more tags with a mass difference of 6.3 mDa.

Shortly after publication of the first‐generation duplex TMT, 4‐plex iTRAQ was reported, designed based on the principles of the original TMT tag (Mertins et al., 2012; Ross et al., 2004) (Figure 8). To further improve multiplexing capacity, a larger balancer group was incorporated in the structure of the 8‐plex iTRAQ (Aggarwal & Yadav, 2016; Choe et al., 2007). However, compared to the 4‐plex iTRAQ, lower protein and peptide identification rates were reported, which might be caused by formation of sequence‐uninformative fragment ions derived from additional internal fragmentation of the larger tag itself (Frost, Greer, Li, 2015; McAlister et al., 2012; Ow et al., 2009; Pichler et al., 2010; Werner et al., 2012).

Among the commercially available reporter‐ion‐based, isobaric tags, the TMT series has gained in popularity in recent years (Dayon & Affolter, 2020), most likely because of its continuously developing multiplexing capabilities (Dayon et al., 2008; Li et al., 2020; McAlister et al., 2012).

#### 3.1.2 DiLeu, DiART, and IBT tags

Since iTRAQ and TMT tags require complex, multistep syntheses with moderate to low yields (Frost, Greer, Li, 2015; Zeng & Li, 2009), several isobaric tags have been prepared as alternatives, such as the N,N‐dimethyl leucine (DiLeu) (Frost et al., 2020; Frost, Greer, Li, 2015; Xiang et al., 2010), the deuterium isobaric amine reactive tag (DiART) (Zeng & Li, 2009; Zhang et al., 2010) and the isobaric tags (IBT) (Ren et al., 2018).

**Figure 10:**
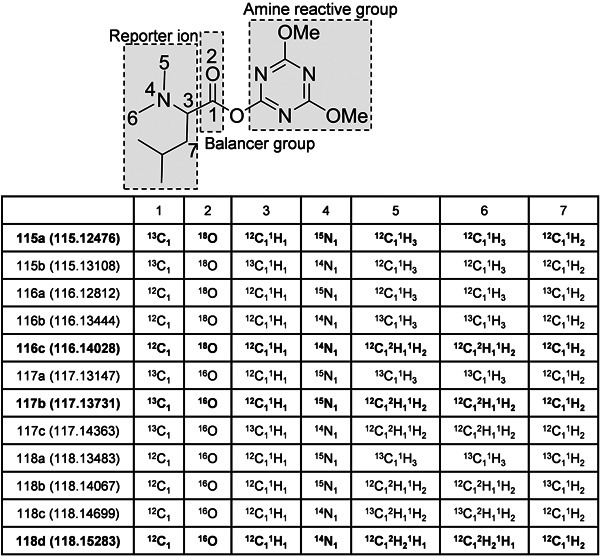
The structure and isotope distribution of 4 and 12‐plex DiLeu. The 4‐plex DiLeu consists of 115a, 116c, 117b, and 118d (marked in bold)

**Figure 11:**
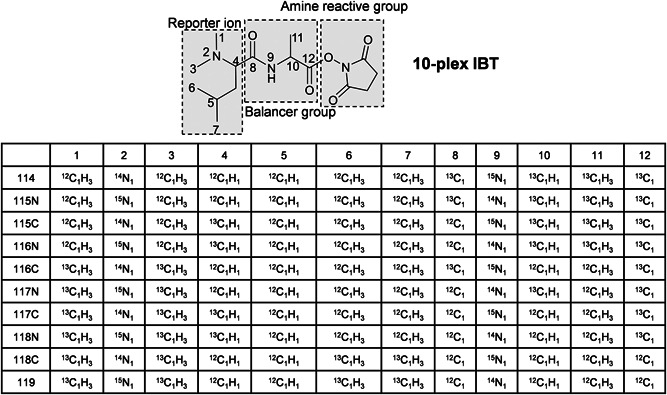
The structure and isotope distribution of 10‐plex IBT. ^13^C and ^15^N are marked with an asterisk

Inspired by the formation of the intense a_1_ ion in dimethylated peptides (Colzani et al., 2008; Fu & Li, 2005; Hsu et al., 2005), Xiang et al. (2010) proposed the 4‐plex DiLeu tag, which has a dimethylated Leu as the core structure, as shown in Figure 10. Essentially, DiLeu is based on the widely used dimethylation strategy in MS1‐based quantification (Hsu et al., 2003) adapted for MS2‐based isobaric labeling. DiLeu‐labeling was reported to give better fragmentation of the peptide backbone and more intense reporter ions when compared to iTRAQ‐labeled peptides (Xiang et al., 2010). The DiLeu tag has only a few carbon or nitrogen atoms and deuterium labels were therefore also incorporated to extend the multiplexing capacity, despite the fact that deuterium labeling affects the retention time of the labeled peptides. This effect was, however, minimized by placing the deuterium atoms in the hydrophilic dimethylamine group, which presumably has less interaction with the reversed‐phase stationary phase (Boersema et al., 2008; Hsu et al., 2003; Zhang et al., 2002). While the deuterium‐induced retention time shift constitutes a disadvantage for this isobaric tag, the commercial availability of different isotopic forms of Leu, the rather straightforward synthesis and the intense reporter ion make DiLeu a valuable alternative to TMT or iTRAQ. Similar to the development of TMT, neutron encoding was employed to increase the multiplexing capacity of DiLeu from 4‐ to 12‐plex (Figure 10), however, with a small mass difference of 5.8 mDa between the reporter ions (Frost, Greer, Li, 2015). By further reducing the mass difference between the reporter ions to 3 mDa by stepwise dimethylation and neutron encoding, the multiplexing capacity of DiLeu was recently improved to 21‐plex without expanding the compact structure of the tag (Frost et al., 2020). However, the required resolution to discriminate this tiny mass difference increases from 30k to 60k (at 400 *m*/*z* for Orbitrap mass analyzers) thus requiring longer cycle times. Instead of relying on neutron encoding, some dimethylated Leu‐based tags have been described that increase multiplexing capacity by incorporating a larger balancer group to replace the carbonyl group in 4‐plex DiLeu, which can only accommodate two isotopes (^13^C and ^18^O). For example, 6‐plex DiART (Zeng & Li, 2009; Zhang et al., 2010) incorporates a β‐alanine and both 8‐plex DiLeu (Frost, Greer, Xiang, et al., 2015) and 10‐plex IBT (Ren et al., 2018) (Figure 11) use an alanine as the balancer between the dimethylated leucine and the amine reactive group. In contrast to 4‐plex or 12‐plex DiLeu, peptides labeled with 8‐plex DiLeu and DiART exhibited considerable retention time shifts between labeling channels due to the more hydrophilic deuterium being inserted in the balancer group (alanine or  β‐alanine), which constitutes a disadvantage for an isobaric, reporter‐ion‐based tag (Frost, Greer, Xiang, et al., 2015). As variations on the same theme, DiAla and DiVal tags have also been synthesized and compared to the DiLeu tag (Yu et al., 2016). Currently, the IBT tag is the only isobaric tag, apart from TMT, that achieves 10‐plex labeling using only ^13^C and ^15^N isotopes.

#### 3.1.3 Novel designs and applications of reporter‐ion‐based tags

**Figure 12:**
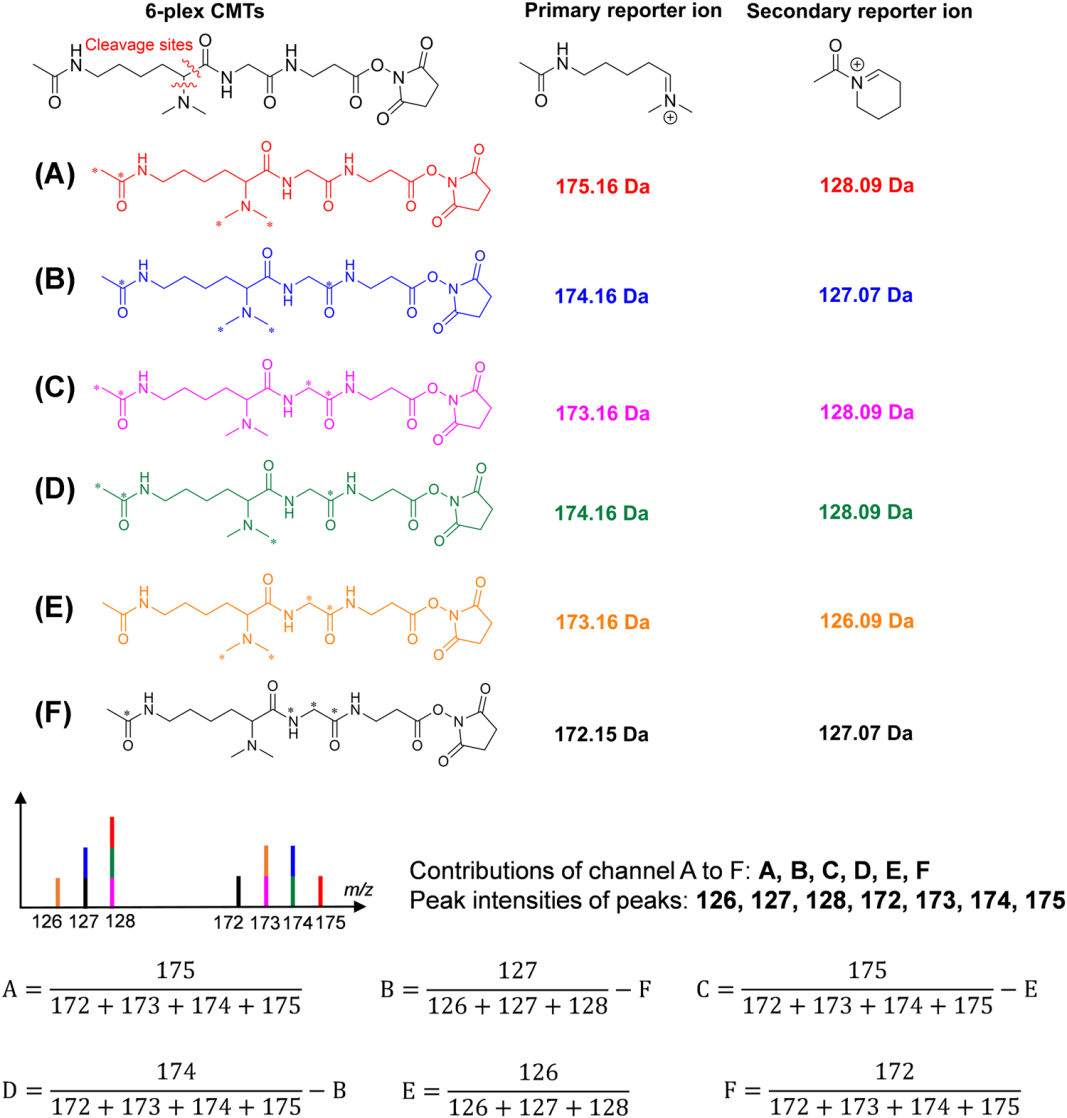
Structure of CMTs and reporter ions. Isotope distribution of 6‐plex CMTs and the principle of reporter ion deconvolution. ^13^C and ^15^N are marked with an asterisk [Color figure can be viewed at wileyonlinelibrary.com]

Besides the aforementioned approaches for relative quantification of peptides based on reporter‐ions, novel concepts and strategies that provide a blueprint for further developments and a wider range of applications of isobaric tags have been proposed. The combinatorial isobaric mass tags (CMTs; Braun et al., 2015) increase the multiplexing capacity by the concept that every isobaric tag produces two sets of reporter‐ions that are independent from each other and where quantitative information can be inferred from the combination of distinct reporter ions (Figure 12). While only showing the results of 6‐plex labeling, the authors anticipate a 16‐plex labeling capacity with a mass shift of ~1 Da between reporter‐ions and an up to 28‐plex labeling capacity with a mass shift of ~6 mDa using only five heavy isotopes. However, the authors pointed out that the formation of reporter ions was affected by the predicated presence or absence of a mobile proton on the precursor peptide (Wysocki et al., 2000). Although there is room for improvement with respect to the structure of this tag, the concept of combinatorial quantification is promising for increasing the multiplexing capacity of quantitative proteomics.

**Figure 13:**
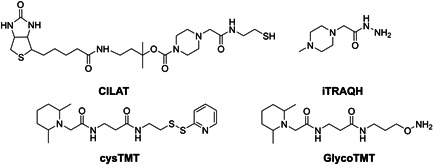
Examples of TMT or iTRAQ based tags for studying glycosylated proteins and proteins containing reactive amino acid derivatives, ortho‐quinone in the oxidized Tyr residue and thiol group of Cys residue

In another development, a series of quantitative approaches targeting specific subclasses of peptides or proteins was reported (Figure 13), namely the cleavable isobaric labeled affinity tag (Li & Zeng, 2007) containing a thiol group targeting the ortho‐quinone produced by oxidation of Tyr residues with tyrosinase, the cysTMT tag targeting Cys residues (Bąchor et al., 2019; Murray et al., 2012), and the GlycoTMT tag (Hahne et al., 2012) and the iTRAQ hydrazide (iTRAQH) (Palmese et al., 2012) targeting reactive aldehyde groups of carbohydrates after oxidation of cis‐diols with periodate. The above labels target specific residues or reactive sites other than primary amine groups, such as cysTMT targeting thiol groups in Cys residues with iodoacetamide analogs or GlycoTMT targeting aldehydes in glycans with aminooxyl analogs. This is especially useful for studying post translation modifications (PTMs), since PTM‐containing peptides are often low abundant and a large amount of starting material is required for MS detection. Accordingly, when using standard isobaric tags to analyze PTMs, a large amount of tag is needed for complete labeling, while the selective reaction strategies reduce the required amount.

#### 3.1.4 **Strategies to correct for ratio distor**tion

**Figure 14:**
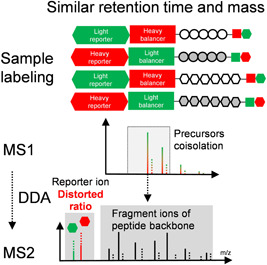
Ratio distortion caused by cofragmentation of multiple peptide precursor ions [Color figure can be viewed at wileyonlinelibrary.com]

While reporter‐ion‐based quantitative approaches exhibit excellent throughput due to their high multiplexing capacity and flexible labeling capability targeting various functional groups, it has become increasingly apparent that reporter‐ion‐based quantification suffers from ratio distortion (Ow et al., 2009; Sonnett et al., 2018; Ting et al., 2011; Wenger et al., 2011; Wühr et al., 2012) caused by peptide cofragmentation, as shown in Figure 14. Data acquisition in DDA mode generally utilizes a window of several Thomson (Th) (Couderc et al., 1991) to isolate precursor ions for fragmentation. While isolation and fragmentation of a single precursor ion results in well‐defined MS2 spectra, in which the ratio of reporter ions reflects the ratio of a given peptide in the differentially labeled samples, this is no longer the case when precursor ions of distinct peptides, that have similar retention times and a mass difference that is smaller than the isolation window, are coisolated. As a result, the produced reporter ions from different peptides are indistinguishable and their intensities no longer reflect the ratios between the peptides in the different samples thus leading to incorrect protein ratios. This issue is more serious for more complex samples and depends further on the efficiency of the chromatographic separation. Although only a minority of peptides and proteins are generally affected, ratio distortion decreases the reliability of the entire analysis as it affects protein ratios in an unpredictable way (Ting et al., 2011; Wühr et al., 2012). In the past decade, a number of approaches have been reported to tackle the ratio distortion problem. They can be classified into the following categories: (1) extending the LC gradient in combination with prefractionation (Ow et al., 2011; Ting et al., 2011), (2) narrowing the precursor isolation window (Savitski et al., 2011; Sonnett et al., 2018; Ting et al., 2011; Winter et al., 2018), (3) delayed fragmentation at the apex of the LC peak (Savitski et al., 2011), (4) additional gas phase reactions (Wenger et al., 2011), (5) subjecting primary fragment ions to an additional isolation and MS3 fragmentation step (Dayon et al., 2012; McAlister et al., 2014; Schweppe et al., 2020; Ting et al., 2011), (6) ion mobility separation to separate coeluting peptides (Pfammatter et al., 2016, 2018; Schweppe et al., 2019), and (7) correcting ratio distortion by estimating the extent of interference or by detecting and discarding MS2 spectra that are produced from multiple precursor ions (Iwasaki et al., 2019; Li et al., 2014).

According to Ting et al. (2011) and Wenger et al. (2011), prefractionation and narrowing the isolation window reduce the problem of ratio distortion only to a limited extent, even though this is somewhat surprising considering that the chance of having peptides of similar *m*/*z* coelute should decrease. This indicates that we are still only scratching the surface of the depth of the peptide mixtures in shotgun proteomics. A delayed fragmentation scheme combined with a narrowed isolation window on Orbitrap instruments was applied to alleviate ratio distortion by 32%, because fragmentation was triggered closer to the apex of a chromatographic peak. This avoided cofragmentation to some extent and afforded better S/N ratios resulting in higher quality MS2 spectra (Savitski et al., 2011). Reducing the average peptide charge state by proton transfer ion‐ion reactions in the so‐called QuantMode method increases the *m*/*z* difference between peptides, thereby reducing the possibility of cofragmentation (Wenger et al., 2011), for example by avoiding interference between a doubly charged precursor ion and another triply charged precursor ion. However, this method is not widely used, since it relies on scan routines that are difficult to optimize and control and that require advanced instrumentation.

In the initially reported MS3‐based approach to correct for ratio distortion, the most intense fragment ion in the MS2 spectrum is selected for further fragmentation. Since b‐ and y‐ions of overlapping precursors are less likely to overlap as well, the resulting reporter ions are less prone to be affected by ratio distortion (Ting et al., 2011). Limited to the isolation of only one fragment ion for secondary fragmentation, the first generation of MS3‐based methods suffered from modest intensities of reporter‐ions in the MS3 spectra. In light of this, the improved MultiNotch MS3 method synchronously isolates multiple fragment ions for secondary fragmentation (synchronous precursor selection, SPS), which increased the intensities of the reporter ions in the MS3 spectra by more than 10‐fold (McAlister et al., 2014), significantly improving the dynamic range and reducing reporter ion signal variance. Nevertheless, only intensity‐based selection is employed which means that selecting fragment ions derived from multiple precursors cannot be avoided. The MultiNotch MS3 method was implemented on an Orbitrap Fusion mass spectrometer, which has an ion trap as well as a dedicated collision cell for multistage fragmentation. Since an additional scan event is required, MS3‐based approaches have longer DDA cycle times and as a result fewer protein identifications in comparison to MS2‐based quantitative methods. Recently, a real‐time database search platform, Orbiter, has been reported to shorten the cycle time by canceling the SPS MS3 scan when there are no peptide matches for a given MS2 spectrum (Schweppe et al., 2020). Compared to the previous MultiNotch MS3 method, the real‐time database search‐based SPS MS3 achieved a twofold faster acquisition rate resulting in more quantified proteins.

High field asymmetric waveform ion mobility spectrometry (FAIMS) is an atmospheric pressure ion mobility technique that separates gas‐phase ions based on their behavior in strong and weak electric fields. FAIMS is easily interfaced with electrospray ionization and has been widely used as a means of on‐line fractionation. More details can be found in the following reviews and publications (Barnett et al., 2002; Bekker‐Jensen et al., 2020; Hebert et al., 2018; Swearingen & Moritz, 2012). Pfammatter et al. utilized FAIMS to reduce ratio distortion by adding a separation dimension based on ion mobility to decrease the chance of precursor ion cofragmentation (Bonneil et al., 2015; Pfammatter et al., 2016, 2018; Sturm et al., 2014). Indeed, FAIMS was shown to robustly improve TMT‐based quantification accuracy and precision without sacrificing peptide/protein identifications (Schweppe et al., 2019).

All of the aforementioned approaches aim at mitigating ratio distortion of reporter‐ions by avoiding peptide cofragmentation during data acquisition. An alternative strategy is postacquisition data processing, such as determining whether precursors were cofragmented based on the elution profiles in LC of the precursor ions. The extent of convolution of precursor ions can then be used to reduce the weighted contribution of chimeric MS2 spectra to the quantification result or to discard a given MS spectrum (Iwasaki et al., 2019; Li et al., 2014). Although it reduces the number of quantifiable peptides, this type of strategy is still the most straightforward, since it does not require any specialized equipment and additional ion separation or fragmentation routines.

### 3.2 Peptide backbone fragment ion‐based quantification

The root cause of ratio distortion in reporter ion‐based methods, such as TMT or iTRAQ, is that the reporter‐ion is not specific for a given peptide. This makes the reporter‐ion unable to accurately reflect the quantitative information once different peptides are cofragmented. To circumvent this pitfall, employing specific fragment ions of the peptide backbone for quantification has been explored. In peptide backbone fragment ion‐based methods, even though distinct peptides labeled with the same labels may be cofragmented, the peptide‐specific fragment ions can still be attributed to the correct peptide thus the quantification information can also be properly assigned.

#### 3.2.1 IPTL‐based labeling strategies

**Figure 15:**
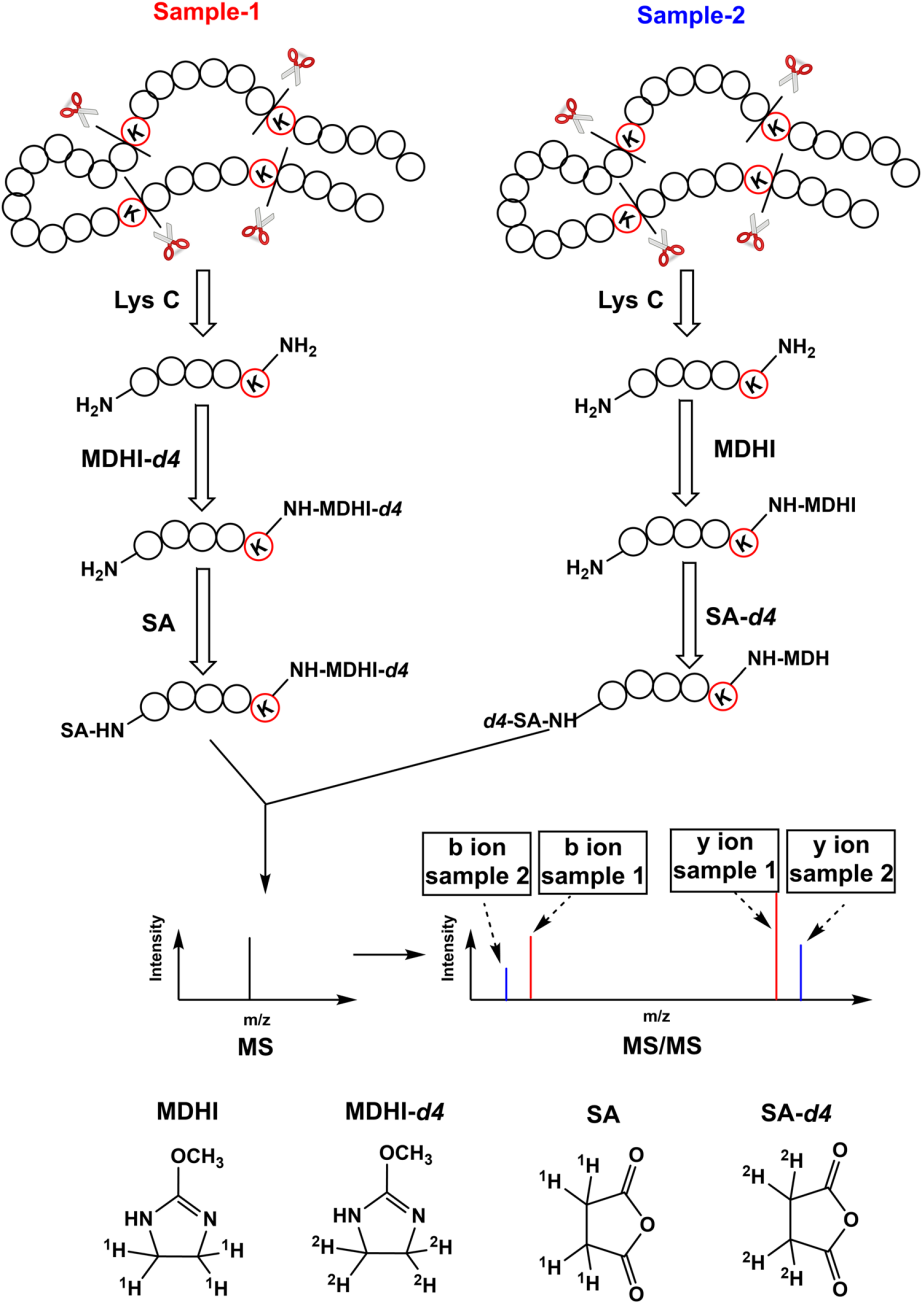
Schematic overview of the original IPTL approach. IPTL, isobaric peptide termini labeling [Color figure can be viewed at wileyonlinelibrary.com]

Koehler et al. (2009) first reported Isobaric Peptide Termini Labeling (IPTL) and proposed the concept of quantifying peptides based on sequence‐specific fragment ions. The original IPTL method used 2‐methoxy‐4,5‐dihydro‐1*H*‐imidazole (MDHI) and the tetradeuterated form 2‐methoxy‐4,5‐dihydro‐1*H*‐imidazol‐4,4,5,5‐*d*_4_ (MDHI‐*d*_4_) to specifically modify the epsilon‐amine group of Lys followed by derivatization of the N‐terminal amine group with succinic anhydride (SA) or tetradeuterated succinic anhydride‐*d*4 (SA‐*d*4) (Figure 15). As a result, the peptides, derived from two samples, are differentially but isobarically labeled, but generate sets of distinct fragment ions upon fragmentation. Since N‐ and C‐termini contain different labels, the peptide and protein ratios can be inferred by comparing the intensities of individual y‐ and b‐series fragment ions. Even in the case that more than one peptide is selected in the same precursor isolation window, the resulting b‐ and y‐ions can usually be correctly attributed to the corresponding peptide. IPTL potentially permits more accurate and precise quantification than reporter‐ion‐based methods due to multiple quantification data points per spectrum, for each y‐ and b‐ion. IPTL does not refer to a label with a specific structure, but rather to a labeling strategy that can achieve isobaric labeling by modifying peptide termini. Based on this principle, a considerable number of methods and applications have been reported in the past ten years by combining various isotope labeling methods, such as rapid‐IPTL (Koehler et al., 2011), triplex‐IPTL (Koehler et al., 2013), and triplex‐QITL (Jiang et al., 2018) based on selective succinylation and dimethylation, IVTAL (Nie et al., 2011), G‐IVTL (Xie et al., 2014), QITL (Yang et al., 2012), diDO‐IPTL (Waldbauer et al., 2017) based on SILAC (Cao et al., 2017; Xie et al., 2014) and proteolytic ^18^O labeling (Waldbauer et al., 2017; Yang et al., 2012) or pIDL (Zhou et al., 2013), PITL (Zhang, Wu, Shan, et al., 2015), and SWATH‐pseudo‐IPTL (Zhang et al., 2016) based on so‐called pseudoisobaric dimethyl labeling. Even though many methods have been reported, the IPTL‐based approaches are not as extensively used as the reporter‐ion‐based approaches, presumably for two reasons: (1) their limited multiplexing capacity, especially when avoiding deuterium labeling and (2) the increasing complexity of MS2 spectra with increasing levels of multiplexing due to multiplets of fragment ions.

#### 3.2.2 Novel IPTL designs with higher multiplexing capacity

**Figure 16:**
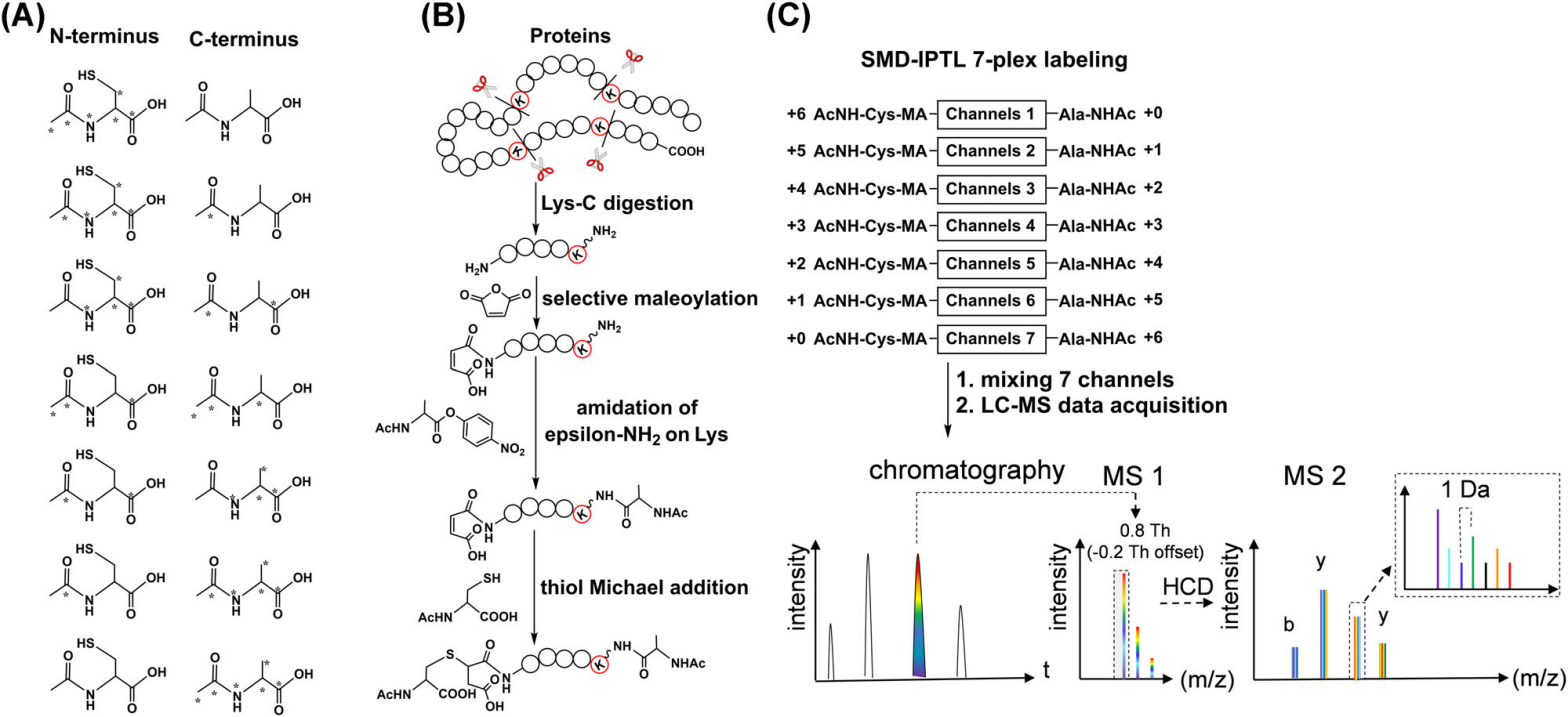
Scheme of SMD‐IPTL. (A) The seven possible combinations of isotopically labeled acetylcysteine and acetylalanine. The atom marked with asterisk denotes ^13^C or ^15^N. (B) Work‐flow of sample preparation and labeling steps of SMD‐IPTL. (C) LC‐MS process for a mixture of 7‐plex labeled samples [Color figure can be viewed at wileyonlinelibrary.com]

Most the above‐mentioned IPTL‐based methods can achieve multiplexing up to triplex, which is far less than for reporter‐ion‐based methods (e.g., TMT has recently been extended to 16‐plex labeling in a single LC‐MS run (Thompson et al., 2019)). Recently, Liu et al. (Liu et al., 2019) reported the so‐called pseudo‐isobaric dimethyl labeling (m‐pIDL) method, which increases the multiplexing capacity to 6‐plex. m‐pIDL relies on a wide isolation window of 10 Th, which increases the complexity of MS2 spectra even further. Furthermore, the use of deuterium in the tags carries the risk of varying the retention time of labeled peptides and may lead to inaccurate quantification. To improve the IPTL multiplexing capacity with non‐deuterium tags, we recently proposed the selective maleylation‐directed isobaric peptide termini labeling (SMD‐IPTL) method (Tian, de Vries, Visscher, et al., 2020) that not only retains all the advantages of the IPTL approaches but also improves the multiplexing capacity to at least 4‐plex labeling with the potential to be extended to 7‐plex labeling by using ^13^C or ^15^N‐labeled cysteine, alanine and acetic anhydride (Figure 16A). SMD‐IPTL is based on the selective maleylation at the N‐termini of LysC peptides. The newly introduced maleyl derivatives can be further modified with different isotopic forms of acetylcysteine, while at the C‐termini the complementary isotopically labeled acetylalanine is inserted to balance the overall mass of the modified peptide, as shown in Figure 16B. Moreover, a precursor ion isolation window of 0.8 Th with an offset of −0.2 Th was used during data acquisition to simplify the isotopic envelopes of fragment ions thus helping in deducing the fragment ion ratios (Figure 16C).

#### 3.2.3 Novel IPTL designs with simpler MS2 spectra

**Figure 17:**
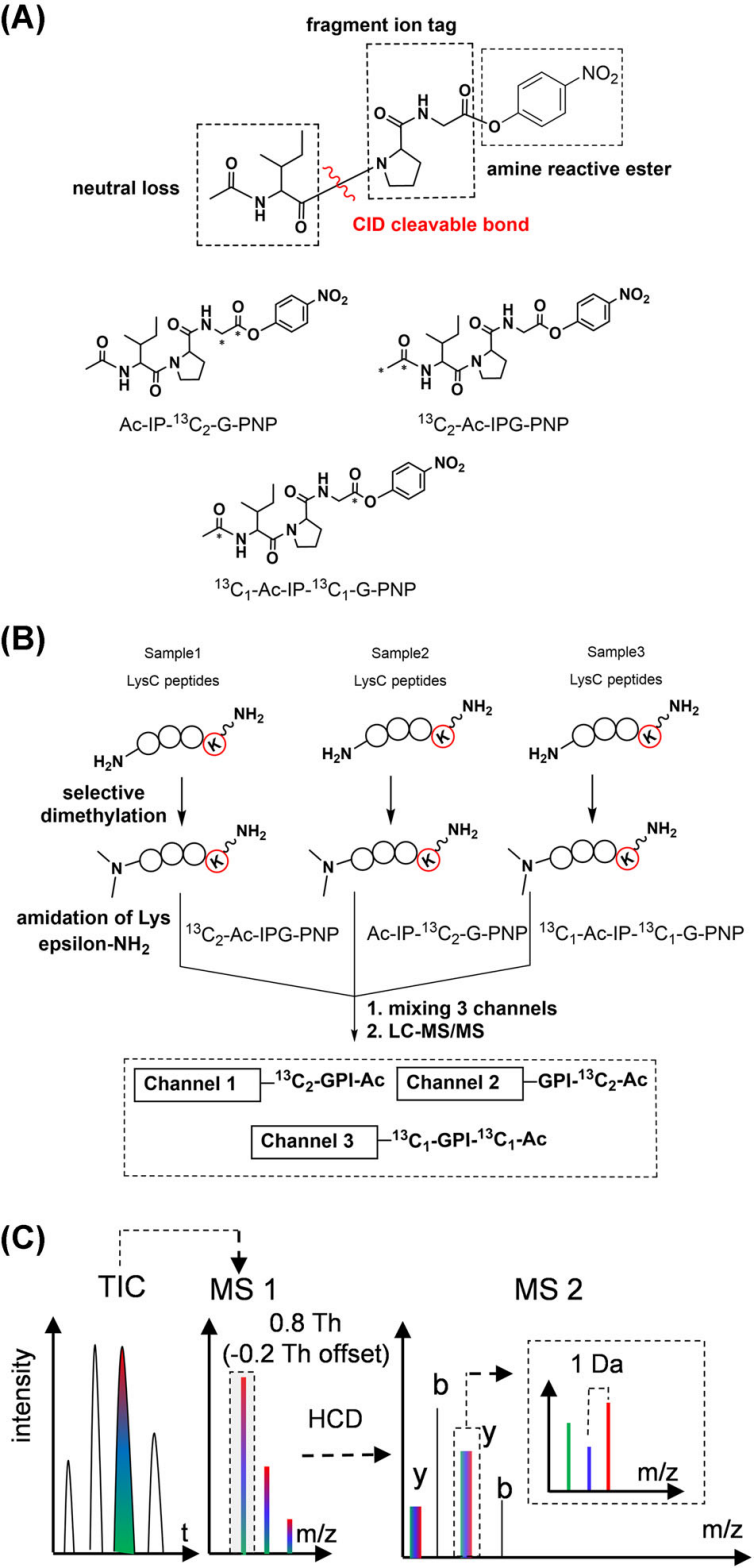
Schematic view of the Ac‐IPG approach. (A) Functional design of the Ac‐IPG‐PNP tag (^13^C isotope locations of the triplex Ac‐IPG‐PNP tag are marked with asterisk); (B) triplex isobaric labeling steps; (C) LC‐MS for a mixture of triplex labeled samples. LC‐MS, liquid chromatography mass spectrometry [Color figure can be viewed at wileyonlinelibrary.com]

Although IPTL methods have advantages compared to reporter‐ion‐based approaches with respect to ratio distortion and quantification accuracy, there is still room for improvement with respect to peptide identification, which is a premise to accurate quantification. In MS2 spectra of IPTL experiments, the number of fragment ions is multiplied by the number of labeled samples, which provides more data points for quantification, but renders identification more challenging. To conserve identification and retrieve accurate quantification information from IPTL data, various software solutions have been reported, such as IsobariQ (Arntzen et al., 2011; Koehler et al., 2011), ITMSQ (Xie et al., 2015), and PISA (Zhang, Wu, Shan, et al., 2015). Peptide identification from IPTL data would be facilitated if the multiplicity of the MS2 fragment ion spectra would not scale with the level of multiplexing. To this end, we developed a collision‐induced dissociation (CID)‐cleavable, isobaric acetyl‐isoleucine‐proline‐glycine (Ac‐IPG) tag (see Figure 17A) (Tian et al., 2020a). The Ac‐IPG tag is based on selective N‐terminal dimethylation (Koehler et al., 2013; Qin et al., 2012) followed by derivatization of the epsilon‐amine group at the C‐terminal Lys residue of LysC peptides with isobaric Ac‐IPG tags having complementary isotope distributions on Pro‐Gly and Ac‐Ile, as shown in Figure 17B. Fragmentation occurs between Ile and Pro (Dongre et al., 1996; Hogan & McLuckey, 2003; Huang et al., 2004; Mák et al., 1998; Tiwary et al., 2019) in addition to fragmentation of the peptide backbone upon CID. While the resulting y‐ions can be distinguished between labeling channels based on the distinct isotopes on the Pro‐Gly part and thus contain the quantitative information for the respective peptides, b‐ions of the different labeling channels have identical *m/z* values, which allows database searching with commonly used algorithms (see Figure 17C). The Ac‐IPG tag conserves the merits of quantifying peptides based on specific peptide fragment ions while reducing the complexity of MS2 spectra compared to conventional IPTL methods thus facilitating peptide identification. The Ac‐IPG tag was used for triplex labeling, but the multiplexing capacity can be extended to 10‐plex with ^13^C‐ and ^15^N‐labeled acetic anhydride, isoleucine, proline and glycine via the reported synthesis route (Tian et al., 2020a).

### 3.3 Peptide‐coupled reporter‐ion based quantification

In reporter‐ion‐based approaches, such as TMT, different peptides derived from the same sample are labeled with the identical tag, which releases indistinguishable reporter ions upon tandem MS, with the associated risk of ratio distortion upon cofragmentation. It is worth noting that the remaining part termed the “peptide‐coupled reporter‐ion,” contains the complete peptide sequence of the precursor with the balancer group attached and thus comprises the complementary quantitative information to the reporter ion (see Figure 1). In contrast to the reporter‐ions, peptide‐coupled reporter‐ions produced from different peptides are peptide‐specific and their mass differences between labeling channels can be discriminated in high‐resolution MS2 spectra (e.g., at 17.5k in an Orbitrap). Only for the extreme cases where the difference between peptide‐coupled reporter‐ions is too small to be differentiated at the set MS2 resolution, the quantitative information cannot be accurately deduced from the peptide‐coupled reporter‐ions. Compared to the IPTL‐based approaches, the biggest merit of peptide‐coupled reporter‐ion based quantification is that differentially labeled peptides have both identical precursor masses and identical fragments ions. While reporter‐ion‐based approaches belong to the mainstream of shotgun proteomics to date, there are only a few recent reports on peptide‐coupled reporter‐ion‐based approaches, namely the TMTc (Wühr et al., 2012), TMTc+ (Sonnett et al., 2018), EASI (Winter et al., 2018), and Ac‐AG tags (Tian et al., 2020b). A limitation of peptide‐coupled reporter‐ions is that they are usually located in the range between *m*/*z* 500–1500, which makes it impossible to use neutron encoding for extending the multiplexing capacity, since a resolution of more than 1 million would be needed to resolve the differentially labeled ions.

#### 3.3.1 **TMTc, TMTc**+, **and EASI tags**

Peptide‐coupled reporter‐ion based quantification was first proposed by Wühr et al. (2012) and called TMTc. However, two limitations leading to unsatisfactory quantification were encountered: (1) the modest efficiency of forming peptide‐coupled reporter‐ions and (2) the complicated isotope envelope of the peptide‐coupled reporter‐ions, which required deconvolution to extract the quantitative information. In this first report, the TMT tag, originally designed for the efficient formation of reporter‐ions, was utilized for labeling and an isolation window of 2 Th was used to isolate precursor ions for fragmentation. A more recent implementation namely TMTc+ used a 0.4 Th precursor isolation window to facilitate data processing, since the monoisotopic precursor ion peak was isolated specifically obviating deconvolution of overlapping isotope envelopes from different labeling channels (Sonnett et al., 2018). Due to the original design of the TMT tag, which favors charge sequestration at the dimethylpiperidine ring, the yield of peptide‐coupled reporter‐ion formation was modest. This showed that the peptide‐coupled reporter ion‐based approach requires specifically designed tags.

**Figure 18:**
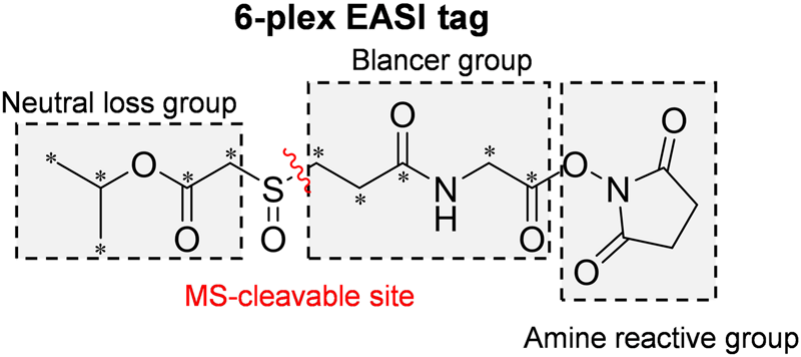
Functional design and isotope distribution of the 6‐plex EASI tag. ^13^C isotope locations are marked with an asterisk [Color figure can be viewed at wileyonlinelibrary.com]

The recently introduced 6‐plex EASI tag (Winter et al., 2018) utilizes a sulfoxide‐based fragmentation site to increase the efficiency of peptide‐coupled reporter‐ion formation and applies an asymmetric isolation window (0.4 Th and −0.15 Th offset) to simplify the isotope envelope of peptide‐coupled reporter‐ions. As shown in Figure 18, the EASI tag consists of a neutral loss group, a balancer group and an amine‐reactive NHS ester. Design of the EASI tag is based on previous work showing that the C‐S bond adjacent to the sulfoxide group fragments easily at low normalized collision energy (NCE) without fragmentation of the peptide backbone (Kao et al., 2011; Stadlmeier et al., 2018). Fragment ions of the peptide backbone are subsequently produced at higher collision energy. Facilitated by the enhanced peptide‐coupled reporter‐ions, EASI tag achieved significant improvements in terms of sensitivity leading to better quantification accuracy and precision.

#### 3.3.2 Ac‐AG tag

**Figure 19:**
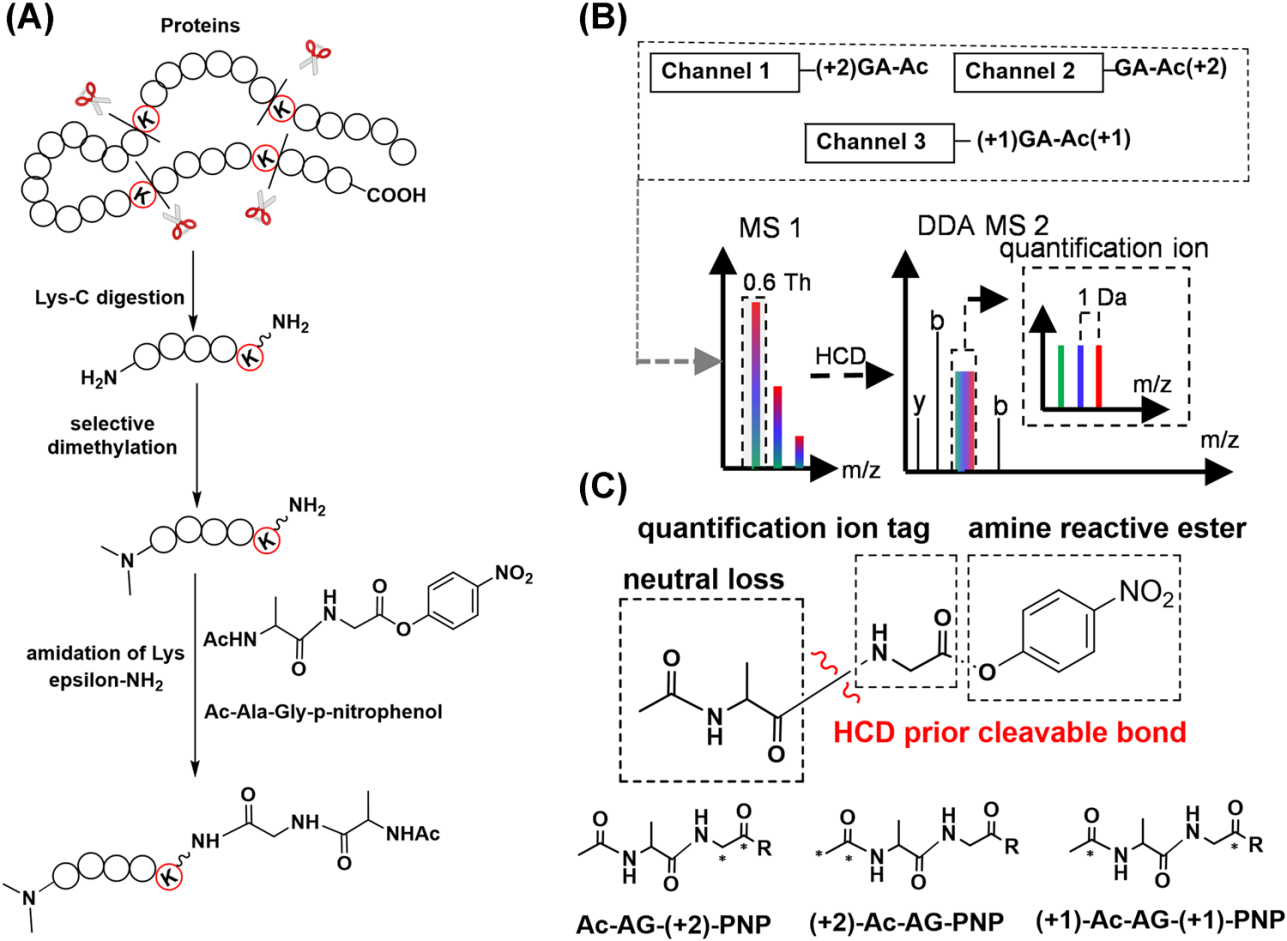
Schematic view of the Ac‐AG approach. (A) Isobaric labeling steps; (B) LC‐MS of a mixture of triplex‐labeled samples in DDA mode; (C) Functional design of the triplex‐labeled Ac‐AG‐PNP tag (^13^C isotope locations are marked with asterisk) [Color figure can be viewed at wileyonlinelibrary.com]

An alternative to EASI tag, the triplex Ac‐AG tag (Tian et al., 2020b) recently reported by our group, is a compact structure consisting of three parts (Figure 19C): (1) Ac‐Ala, which will form a neutral loss upon fragmentation between the Ala and Gly residues; (2) the Gly part, which will remain on the peptide‐coupled reporter‐ion; and (3) an amine reactive p‐nitrophenol ester (PNP). Complementary isotope distribution is designed between the Ac‐Ala and the Gly parts, so that different forms of the Ac‐AG tag are isobaric. To enhance ionization efficiency and promote the formation of b‐ions as well as to block the N‐terminal amine group, the N‐terminal amine groups are first dimethylated before coupling the Ac‐AG tag to the epsilon‐amine group of C‐terminal Lys residue of LysC peptides. The key feature of the tag is that the bond between Ac‐Ala and Gly fragments before fragmentation of the peptide backbone at a low NCE generating an intense peptide‐coupled reporter‐ion while fragment ions of the peptide backbone are generated at a higher NCE. By combining two NCEs in the same MS/MS scan, the ions required for both identification and quantification are acquired in the same MS2 spectrum. Since there is no isotopic label at the N‐terminus and y‐ions contain the entire Ac‐AG tag, all fragment ions of the peptide backbone originating from different labeling channels have the same respective masses, which means that the complexity of MS2 spectra does not increase with the number of differentially labeled samples. Since Ac‐Ala does not have a good ionization site (Figure 19A), it is lost as a neutral molecule and the peptide‐coupled reporter‐ion with the attached Gly part from the Ac‐AG tag has the same charge state as the precursor ion (Tian et al., 2020b; Winter et al., 2018). A narrow precursor isolation window of 0.6 Th was used to simplify the isotope envelops of the peptide‐coupled reporter‐ion, as shown in Figure 19B.

### 3.4 Summary of MS2‐based quantification

To conclude, the most widely used quantification strategy based on isotope labeling is isobaric labeling‐based MS2 quantification using reporter ions. From the first TMT approach proposed in 2003 to the recently reported TMTpro (Li et al., 2020; Thompson et al., 2003), the isobaric labeling capacity has increased eight times from duplex to 16‐plex with neutron encoding (McAlister et al., 2012). During this time remedies such as MS3‐based quantification (Ting et al., 2011), ion mobility‐based quantification (Pfammatter et al., 2016) and peptide‐coupled reporter ion‐based quantification (Wühr et al., 2012) have been proposed to circumvent the problem of ratio distortion of reporter ion‐based approaches. Quantification based on isobaric labeling has become an indispensable strategy in quantitative proteomics, due to its excellent throughput, precision and accuracy. Readers are referred to a recent, more application‐oriented review with respect to the use of chemical labels in quantitative proteomics (Liu et al., 2020).

## 4 COMBINED MS1 AND MS2‐BASED QUANTIFICATION

Driven by the need for a higher multiplexing capacity, hybrid labeling strategies combining isotopic and isobaric labeling in the same sample preparation work‐flow have been developed to increase the throughput of isobaric labeling approaches by two to three times (Evans & Robinson, 2013). The basic idea is to use the mass difference at the MS1 level to further parallelize the analysis of two or three sets of isobarically labeled samples in a single LC‐MS run. Published approaches, comprise the 18‐plex Hyperplexing (Dephoure & Gygi, 2012) (triplex SILAC combined with 6‐plex TMT), 12‐plex cPILOT (Evans & Robinson, 2013) (duplex N‐terminal selective dimethylation combined with 6‐plex TMT) and 24‐plex DiLeu cPILOT (Frost et al., 2018) (duplex N‐terminal selective dimethylation combined with 12‐plex DiLeu), metabolically or chemically incorporate distinct isotopic labels followed by chemical derivatization with isobaric tags. As these strategies are all based on the orthogonal combination of MS1 and MS2‐based quantification strategies, they inherently suffer from the issues of MS1‐ and MS2‐based quantification (Aggarwal et al., 2019), such as the multiplied complexity of spectra at the MS1 level and ratio distortion caused by peptide cofragmentation at the MS2 level. It should be pointed out that, for a given LC‐MS platform, more complex samples are more seriously affected by these limitations.

## 5 ISOTOPE LABELING‐BASED QUANTIFICATION IN DIA MODE

As discussed in Section 3, increasing the tag size (Pierce et al., 2008) and neutron‐encoding (Werner et al., 2012) were applied to increase the throughput of isobaric labeling in DDA up to a maximum capacity of analyzing 16 isobarically labeled samples in a single LC‐MS run (Thompson et al., 2019). However, the stochastic nature of selecting precursor ions for tandem MS in DDA leads to an imperfect overlap in peptide identifications, which means that there is a considerable likelihood that some peptides might be missed in some runs depending on their relative abundance in a given sample (Brenes et al., 2019). Since only the peptides that are measured in all runs can be reliably compared across many runs in studies comprising hundreds or more samples, it would be beneficial to use a strategy that does not rely on data‐dependent and thus stochastic precursor ion selection. This has led to the development of DIA modes of operation, where all precursor ions in a predefined *m*/*z* window are fragmented notwithstanding their relative intensity in the MS1 spectrum.

DIA approaches have gained in popularity notably in large‐scale biomedical studies, for example related to clinical biomarker discovery, as they largely avoid the missing value problem of DDA. Another advantage of DIA is that a so‐called digital fingerprint of a sample is acquired that can later be interrogated for novel features (e.g., biomarker candidates) as new insights emerge from biology without the need to analyze the sample again.

A severe drawback of DIA is, however, that most of the current DIA‐based approaches can only analyze one sample per LC‐MS run, which limits throughput. It would thus be ideal to combine DIA with the multiplexing capability of DDA. However, multiplexed labeling is rarely used in DIA, presumably because current approaches lead to more complex MS2 spectra, severe ratio distortion, and/or a reduction in quantification accuracy and precision. Reporter ion‐based TMT or iTRAQ approaches are not suitable for DIA due to massive cofragmentation. For isotopic labeling and peptide fragment ion‐based isobaric labeling strategies, such as SILAC (Jiang & English, 2002; Ong et al., 2002) and IPTL (Koehler et al., 2009), the peptides from different labeling channels have sets of distinct fragments ions, which multiplies the complexity of MS2 spectra with the number of labeled samples making identification of peptides extremely challenging, since MS2 spectra of DIA are already highly convoluted. Still, there are initial attempts at developing multiplexed stable isotope labeling strategies for DIA, which are briefly discussed below.

### 5.1 NeuCoDIA, MdFDIA, and mdDiLeu tags

Motivated by the need for higher throughput in DIA, researchers have devised novel labeling strategies. The recently reported DIA multiplexed approaches, NeuCoDIA (Minogue et al., 2015) and MdFDIA (Di et al., 2017) incorporate isotopes during cell culture (metabolic labeling), while the mdDiLeu approach uses chemical labeling (Zhong et al., 2020). They all have the same principle of incorporating a mDa mass difference by neutron encoding in peptides. Upon tandem MS, the multiplets of fragment ions containing the neutron‐encoded tag represent the quantification information, which is comparable to the quantification principle of neutron‐encoded MS1 based quantification discussed in Section 2.4. These methods rely on ultrahigh resolution (>120k in the *m*/*z* 100–1000 range) to discriminate the mDa difference at the fragment ion level with, as a consequence, a reduced data acquisition rate in Orbitrap mass analyzers. Based on the DIA cycle time calculator reported by Scheltema et al. (2014), using 20 isolation windows on an Orbitrap Fusion mass spectrometer gives a cycle time of 2.2–4.3 s at an MS2 resolution of 30k, while the cycle time increases to around 8 s at a resolution of 120k, the lower end for discriminating neutron‐encoded fragment ions. The low scanning rate negatively affects identification and quantification and is by and large not suitable for large‐scale comparative proteomics studies.

### 5.2 Application of Ac‐AG tag in DIA mode

**Figure 20:**
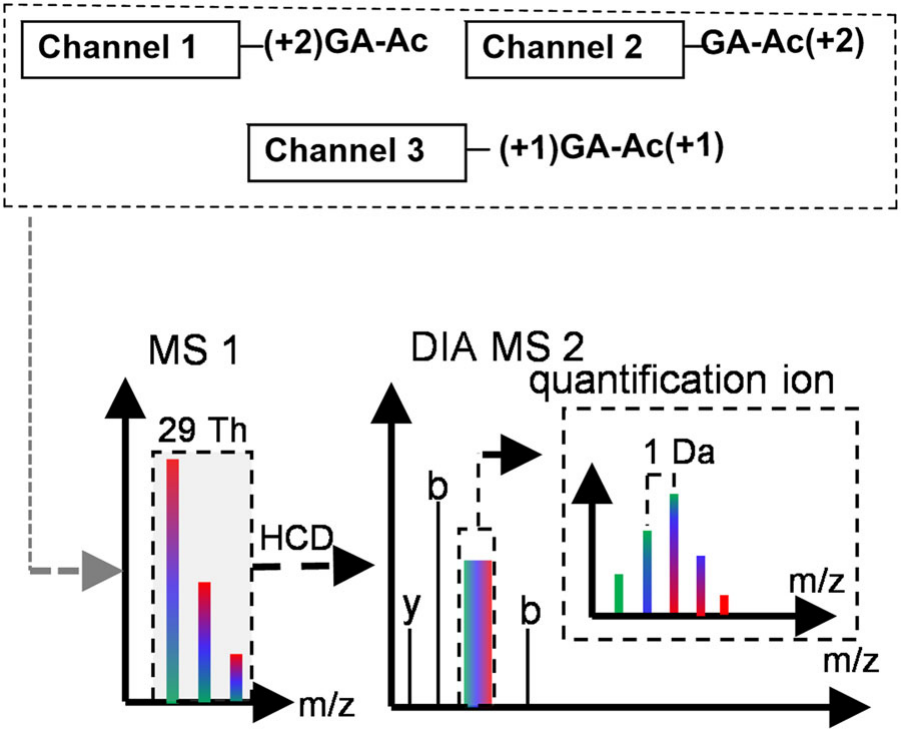
Schematic view of the application of the Ac‐AG tag in the DIA mode. DIA, data‐independent acquisition [Color figure can be viewed at wileyonlinelibrary.com]

To improve the throughput of DIA‐based methods without extending the cycle time and complicating the MS2 spectra, we recently proposed the use of the isobaric Ac‐AG tag (Tian et al., 2020b), that was already discussed in Section 3.3.2, in the DIA mode. The Ac‐AG tag can be applied in both DDA and DIA, because peptides in differentially labeled samples have the same precursor masses, give the same peptide backbone fragment ions, but have different peptide specific peptide‐coupled reporter‐ions that can be discriminated at an MS2 resolution of 20k, which is commonly used in proteomics and available on a wide range of high‐resolution mass spectrometers including time‐of‐flight mass analyzers. While in DDA, the intensities of the peptide‐coupled reporter‐ions simply reflect the quantitative ratios between peptides in the samples that constitute the mixture, in DIA the Ac‐AG tag produces peptide‐coupled reporter‐ions with complete isotope envelopes that overlap for adjacent labeling channels and from which the quantification information can be deduced after deconvolution (Figure 20). It is to be expected that more labeling strategies for multiplex DIA will emerge in the near future and that the required data processing approaches will be developed based on extending existing algorithms.

## 6 SUMMARY

In this review, we discussed the development of stable isotope‐based proteome quantification approaches using chemical labeling and compared their strengths and weaknesses. It took 20 years from the first isotopic tag, ICAT (Gygi et al., 1999), to the currently released most advanced isobaric tag, TMTpro (Thompson et al., 2019). There is still room for optimization and the development of new isotopic and isobaric tags and labeling strategies, notably for DIA. The design of isotopic tags requires, among others, (1) to balance the formation of ions for quantification and fragment ions of the peptide backbone for identification, (2) to avoid formation of sequence uninformative fragment ions, (3) to increase multiplexing capacity, and (4) to limit synthesis cost and practicality. Amongst all discussed methods, the reporter ion‐based MS2 or hybrid quantification methods, such as TMTpro (16‐plex) and DiLeu (21‐plex), have the highest multiplexing capacity. With the combination of MS3‐based quantitation and the assistance of ion mobility spectrometry, the problem of ratio distortion has been tackled although not overcome completely. Increasing multiplexing capacity at a reasonable cost is highly desirable to allow the analysis of large sample numbers, for example, in clinical proteomics studies. We anticipate that the combination of multiplexed labeling and DIA will be more widely used for large‐scale studies in the future. Besides, it should be noted that the widespread usage of a specific tag is not only determined by its technical merits but also by its commercial availability. Last but surely not least, it is important to keep in mind that, besides the labeling strategies discussed here, the advancement of mass spectrometer technology and sophisticated data processing workflows are equally important to advance this field.

## CONFLICT OF INTERESTS

The authors declare that there are no conflicts of interest.
